# Insights into muscle metabolic energetics: Modelling muscle-tendon mechanics and metabolic rates during walking across speeds

**DOI:** 10.1371/journal.pcbi.1012411

**Published:** 2024-09-13

**Authors:** Israel Luis, Maarten Afschrift, Friedl De Groote, Elena M. Gutierrez-Farewik

**Affiliations:** 1 KTH MoveAbility, Dept. Engineering Mechanics, KTH Royal Institute of Technology, Stockholm, Sweden; 2 Faculty of Behavioural and Movement Sciences, VU Amsterdam, Amsterdam, The Netherlands; 3 Department of Movement Sciences, KU Leuven, Leuven, Belgium; 4 Department of Women’s and Children’s Health, Karolinska Institutet, Stockholm, Sweden; University of Birmingham, UNITED KINGDOM OF GREAT BRITAIN AND NORTHERN IRELAND

## Abstract

The metabolic energy rate of individual muscles is impossible to measure without invasive procedures. Prior studies have produced models to predict metabolic rates based on experimental observations of isolated muscle contraction from various species. Such models can provide reliable predictions of metabolic rates in humans if muscle properties and control are accurately modeled. This study aimed to examine how muscle-tendon model individualization and metabolic energy models influenced estimation of muscle-tendon states and time-series metabolic rates, to evaluate the agreement with empirical data, and to provide predictions of the metabolic rate of muscle groups and gait phases across walking speeds. Three-dimensional musculoskeletal simulations with prescribed kinematics and dynamics were performed. An optimal control formulation was used to compute muscle-tendon states with four levels of individualization, ranging from a scaled generic model and muscle controls based on minimal activations, inclusion of calibrated muscle passive forces, personalization of Achilles and quadriceps tendon stiffnesses, to finally informing muscle controls with electromyography. We computed metabolic rates based on existing models. Simulations with calibrated passive forces and personalized tendon stiffness most accurately estimate muscle excitations and fiber lengths. Interestingly, the inclusion of electromyography did not improve our estimates. The whole-body average metabolic cost was better estimated with a subset of metabolic energy models. We estimated metabolic rate peaks near early stance, pre-swing, and initial swing at all walking speeds. Plantarflexors accounted for the highest cost among muscle groups at the preferred speed and were similar to the cost of hip adductors and abductors combined. Also, the swing phase accounted for slightly more than one-quarter of the total cost in a gait cycle, and its relative cost decreased with walking speed. Our prediction might inform the design of assistive devices and rehabilitation treatment. The code and experimental data are available online.

## Author summary

Simulations of the musculoskeletal system can provide insights into motion mechanics and energetics. To increase confidence in the approach, it is important to establish how predictions are affected by modelling assumptions. In this study, we evaluated how simulations with increasing levels of sophistication and metabolic energy models from the literature agreed with recorded muscle excitations, fiber lengths, and whole-body metabolic rates during walking at different speeds. We found that calibrating muscle passive components: muscle passive forces, and tendon stiffness, estimated better muscle excitations and fiber lengths than using original musculoskeletal model values. The metabolic energy cost of walking was closely estimated with only a subset of metabolic energy models. We estimated that the metabolic energy demands are the highest during three periods of the gait cycle—early stance, pre-swing, and initial swing, at all walking speeds. In all walking speeds, muscles that support plantarflexion accounted for the highest metabolic cost among all the muscle groups. As walking speed increases, the metabolic cost of the stance phase also increases. Our detailed description of metabolic costs at muscle groups and gait phases might help to design assistive devices and rehabilitation treatments.

## 1. Introduction

Metabolic rate is a measure of the metabolic demand of muscular effort during motion and basal functions over time. A comprehensive understanding of the metabolic rate can support rehabilitation treatments [1] or inform the design of assistive devices [2,3]. Whole-body average metabolic rate is typically measured indirectly through spiroergometry, whereby oxygen and carbon dioxide rates in inhaled and exhaled breaths are measured as a function of time [4]. This method is used for metabolic assessment in constant (e.g., walking) [1] and non-constant conditions (e.g., sprint and shuttles) [5] after some time span. Measurement of the instantaneous metabolic rate of muscles is impossible due to the slow mitochondrial dynamics, body transit delays, respiratory control mechanisms, and high breath-by-breath variability [6]. Various experimental and computational methods have been developed to estimate metabolic rates from the muscle to the whole-body level.

Metabolic rates of muscles in vivo can be derived from various degrees of experimental observations. Prior studies have examined the mechanics and energetics of a single or few muscles simultaneously during isometric and cyclical contractions using measured muscle excitation, fiber length, and force data [7–9]. On the contrary, state-of-the-art wearable systems can accurately predict instantaneous metabolic rates from segment motions, but only at the whole-body level [10]. An alternative approach to estimating whole-body and individual muscle metabolic rates is using computational methods such as musculoskeletal modelling. Models of muscle dynamics, such as the Hill-type model [11], combined with metabolic energy models [12–14], can be used to estimate muscle-tendon states and metabolic rates.

Multiple metabolic energy models have been developed based on experimental datasets and assumptions of muscle energetics. Several models have been used frequently, including those proposed by Bhargava et al. (BH04) [12], Houdijk et al. (HO06) [15], Lichtwark and Wilson in its original (LW05) and modified version (LW07) [16], Umberger in its initial (UM03) [14] and revised version (UM10) [17], and a further modification proposed by Uchida et al. (UC16) [18] (S1 Table). These models use the same definition of contractile element work rate but different formulations of the heat rate. The total heat rate is primarily composed of four terms: activation rate, associated with non-contractile costs of muscle activation to pump Ca++ ions against a concentration gradient across the sarcoplasmic reticulum; maintenance rate, associated with the cycling of actin-myosin cross-bridges during isometric tetanus; and shortening and lengthening rates, associated with an extra cost of actin-myosin cross-bridge interactions during dynamic muscle contractions.

Metabolic energy models have been reported to predict metabolic rates during walking in people with and without motion disorders and assistive devices with reasonable accuracy. The whole-body average metabolic rate of walking from forward dynamics simulations was estimated as 5.8 W/kg at 1.36 m/s with the BH04 model [12], 4.4 W/kg at 1.2 m/s with the UM03 model [14], ~5 W/kg at 1.3 m/s with the UM10 model [17], and 4 W/kg at 1.46 m/s using the UC16 model [19]. These estimations were relatively close to experimental energy measured with indirect calorimetry, except with the BH04 model, which was higher. Interestingly, the BH04 model accurately estimated metabolic rates in a small clinical population, specifically in two subjects post-stroke across slow walking speeds [20] and in an amputee with a transtibial passive prosthesis at 1.3 m/s [21]. The metabolic rate changes related to using assistive devices in able-body individuals during hopping were estimated with the LW07 model [22] and during walking with the UC16 model [23]. Despite these encouraging findings, evaluating the accuracy of various metabolic energy model formulations is challenging, as they depend on the underlying muscle-tendon states, which are in turn dependent on musculoskeletal models and simulation approaches.

Two previous studies have assessed the accuracy of and differences between metabolic energy models in both forward simulations and inverse dynamic analyses. Miller compared metabolic energy models in tracking and predictive simulations at 1.45 m/s and reported that none predicted the absolute value of (3.35 Jm^-1^kg^-1^) accurately [24]. Their estimates varied from 2.45 Jm^-1^kg^-1^ using the BH04 model to 7.15 Jm^-1^kg^-1^ using the LW07 model. Miller also found that differences in the metabolic energy models are mainly attributed to descriptions of active lengthening and eccentric work. Koelewijn et al. performed 2D musculoskeletal simulations with prescribed kinematics and dynamics and evaluated walking at two speeds and three incline levels [25]. They reported a good correlation between estimated metabolic cost and indirect calorimetry measurements, though the estimated cost tended to underestimate the recorded cost, particularly at higher metabolic demands.

Further insights may be gained by evaluating the metabolic energy models based on as accurate estimations of the muscle-tendon states as possible. Simulations performed by Miller [24] and Koelewijn et al. [25] estimated muscle-tendon conditions by assuming that human motor control in gait is dictated by minimum muscle effort. They validated the estimated muscle control by comparing computed muscle activations to measured electromyography signals (EMGs). Indeed, numerous researchers have used this approach to validate their computed muscle control [26] in lieu of any more precise and practical validation method. However, muscle activations are not linearly related to metabolic rates. Metabolic rates are also dependent on muscle-tendon states; further validation of estimated muscle-tendon states should thus improve the accuracy of metabolic rate estimations. Muscle-tendon mechanics, including tendon compliance [16], muscle passive forces [27], and muscle control [28], play a critical role in muscle forces and states, thereby influencing the metabolic rate of muscles. Increasing the levels of model parameter individualization is expected to yield better estimates of muscle energetics compared to generic values, though, to the best of our knowledge, no such study exists. Analysis of how model parameters influence muscle states, such as muscle control and fiber lengths, can help better understand their relationships with the associated metabolic costs during gait.

In this study, we aimed to evaluate the influence and accuracy of various degrees of muscle-tendon parameter individualization and metabolic energy models on estimated muscle-tendon states and muscle metabolic rates, respectively. Also, based on the simulation with the highest agreement with measured values, we aimed to provide a reliable prediction of the metabolic cost of the gait phases and muscle function groups across walking speeds. We hypothesize that as model individualization increases, the accuracy of the simulated muscle-tendon states and metabolic cost will also increase, as will estimated metabolic rates overall and in different muscles/muscle groups, and at a range of walking speeds.

## 2. Methods

### 2.1. Ethics statement

The study was approved by the Swedish Ethical Review Authority (Dnr. 2020–02311), and all participants provided written consent. Participation was voluntary and could be terminated at any time during the experiment.

### 2.2. Participants

Eight unimpaired adults (5/3 male/female, age: 33.6 ± 8.5 years old, height: 1.74 ± 0.10 m, body mass: 71.3 ± 10.9 kg [mean ± SD]) participated in this study. Our inclusion criteria were participants between 25 and 60 years old with no known pathology or recent injury affecting walking and who could commit to fasting 2.5 hours before data collection with spiroergonometry.

### 2.3. Experimental protocol and data processing

Subjects walked on a treadmill at 55%, 70%, 85%, 100%, 115%, 130%, and 145% of their estimated preferred walking speed (PWS) in two conditions: treadmill, then overground walking, at the Promobilia MoveAbility Lab. The PWS was predetermined by the participant’s gender, age, and height [29]. In treadmill trials, oxygen and carbon dioxide respiration rates were recorded during 6 minutes of walking at each speed (Cortex Metamax 3B, Leipzig, Germany). The representative value from each speed was computed based on the average in the last 3 minutes. After each trial, each subject rested for 5 minutes to avoid fatigue. At each experimental session, speed was randomized, and average cadence was recorded.

In overground walking experiments, participants walked along a lab pathway and emulated different walking speeds by matching recorded cadences from treadmill walking with audio signals from a metronome app. Marker position (100 Hz) and ground reaction forces (1000 Hz) were measured using optical motion capture (Vicon V16, Oxford, UK) and strain gauge force platforms (AMTI, Watertown, MA, USA), respectively. Full-body marker placement was implemented based on the Conventional Gait Model with the Extended-foot model (CGM 2.4). EMG from eight muscles in one leg were recorded: biceps femoris long head, semitendinosus, vastus lateralis and medialis, tibialis anterior, gastrocnemius lateralis and medialis, and soleus, using bipolar surface wireless electrodes (Myon aktos/Cometa systems, Milan, Italy). The selection of sides for EMG placement was randomized. Skin preparation and electrode placement followed the Electromyography for the Non-Invasive Assessment of Muscles guidelines (SENIAM) [30]. Subjects were asked to perform functional tests, including standing heel raise, standing toe raises, squat, knee flexion, and hip flexion/extension, to corroborate EMG placement. EMG signals were processed using a 4th-order zero-lag Butterworth band-pass filter (20–400 Hz), full-wave rectification, and a 4th-order zero-lag Butterworth low-pass filter (6 Hz). EMG of the vastus intermedius was estimated as an average of the vastus lateralis and medialis. Ground reaction forces were processed using a 4th-order zero-lag Butterworth low-pass filter (35 Hz).

### 2.4. Musculoskeletal simulation framework

#### 2.4.1. Musculoskeletal model, scaling method, and inverse kinematics and dynamics

The generic musculoskeletal model developed by Rajagopal et al. [31] was selected for this study. We scaled the generic model using OpenSim’s Scale Tool, which adjusted skeletal geometry and, therefore, changed muscle-tendon lengths, moment arms and wrapping objects, and segment inertial properties to fit anthropometric dimensions obtained from a captured static calibration trial. Also, with this tool, the optimal fiber lengths and tendon slack lengths of each muscle-tendon actuator are scaled linearly to preserve the ratio of the generic model in the scaled model. The maximum isometric force of each muscle-tendon actuator was individualized based on the expected muscle volume, which in turn was estimated based on body weight and height, according to a regression equation proposed by Handsfield et al. [32]. Each muscle’s maximum isometric force is assumed to be directly proportional to its physiological cross-sectional area and specific tension. Muscle active and passive force-length and force-velocity relationships were modeled as described by De Groote et al. [33].

Marker trajectories and ground reaction forces throughout 21 gait cycles per subject (3 cycles at each of 7 walking speeds) were analyzed with inverse kinematics and inverse dynamics using OpenSim 4.1. Marker tracking weights for inverse kinematics were selected to minimize the error between experimental and virtual markers. The subtalar and metatarsal joints were fixed at neutral anatomical positions.

These model and muscle-tendon scaling methods were selected based on a recent study in which we determined that this combination yielded the best agreement between computed fiber lengths and those measured with ultrasound and between computed muscle excitations and measured EMGs in the vastus lateralis and medialis, tibialis anterior, gastrocnemius medialis and lateralis and soleus [34]. In that study, we observed a poor estimation of the activations in the biceps femoris long head and semitendinosus, which we hypothesized to be due to the overestimation of passive tendon forces at knee and hip joints [31,35]. We address this concern in the current study.

#### 2.4.2. Calibrated passive muscle force-length relationships

Parameters of the muscle passive force-length relationships in the generic musculoskeletal model were calibrated to better agree with the passive moment-angle relationship measured experimentally by Silder et al. [36]. The relationship was modeled based on OpenSim’s Thelen2003Muscle as described by Thelen [37] (Eq 1)

$$f_{pas}({\tilde{l}}_{M})=\frac{e^{\frac{k_{PE}({\tilde{l}}_{M}-s_{0})}{s_{M}}}-1}{e^{k_{PE}}-1}$$ (1)

where ${\tilde{l}}_{M}$ is the normalized fiber length, *k*_*PE*_ is the exponential shape factor for the passive force-length relationship, *s*_0_ is the normalized fiber length at which the passive force starts to increase, and *s*_*M*_ is the normalized fiber length, measured from the optimal fiber length (${\tilde{l}}_{M}=1$), at which maximum force is reached. We formulated an optimization routine wherein we selected *k*_*PE*_ and *s*_0_ as optimization variables, with the objective function of minimizing the error between the simulated joint moments from the generic model and the experimental joint moments reported by Silder et al. [36]. The calibrated passive moments closely resembled the measured passive joint moments reported by Silder et al. (S1 Fig). Detailed information about the optimization routine is presented in S1 Appendix (section A: Calibration of passive forces).

#### 2.4.3 Simulation workflow and optimized muscle-tendon parameters

**Fig 1:**
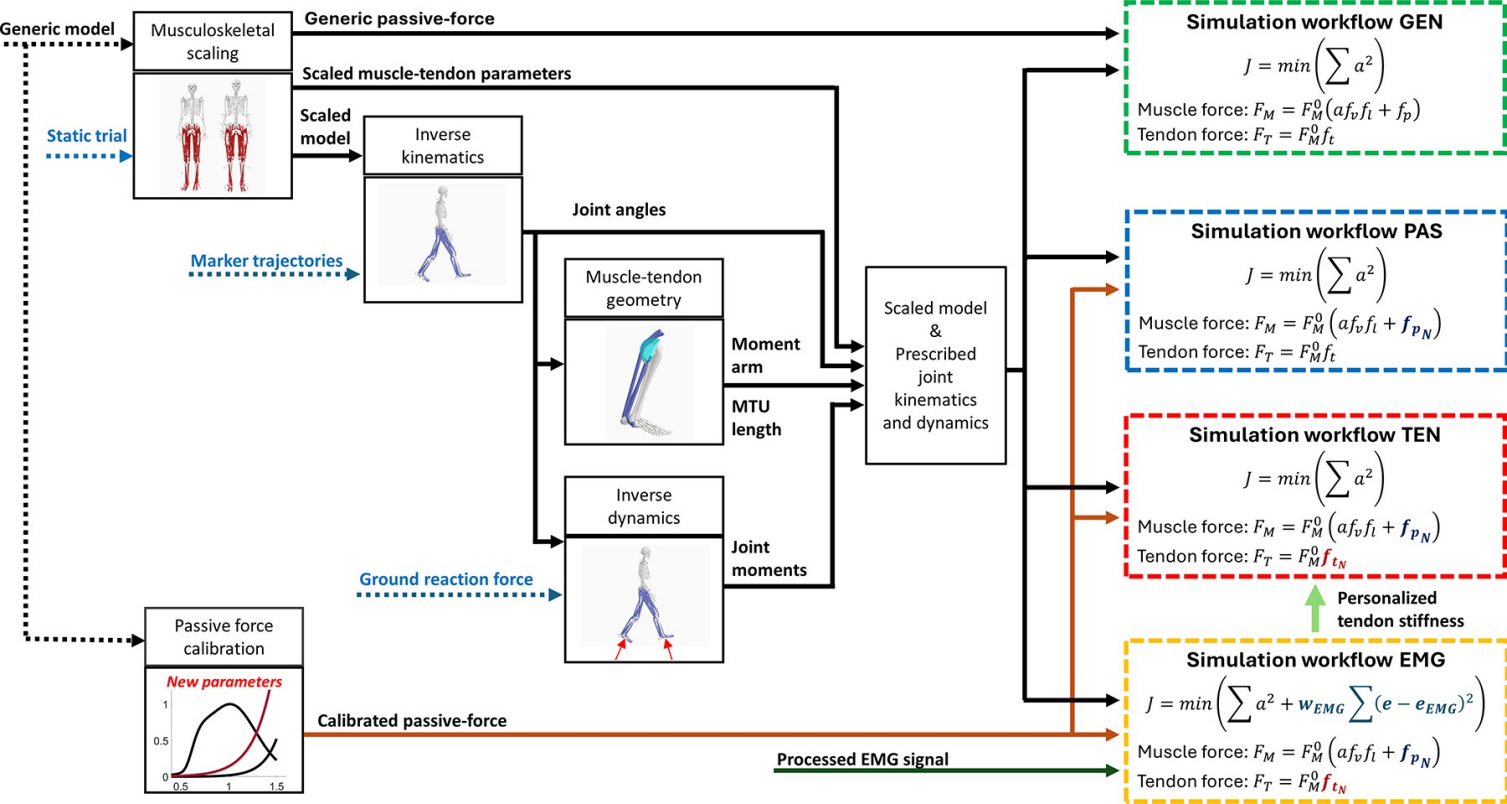
*Diagram of the simulation workflows*. Computed inverse kinematics, inverse dynamics, muscle-tendon length, and moment arms are inputs for all the simulation workflows. In simulation GEN, the performance criterion is the sum of squared muscle activations, the muscle force (*F*_*M*_) is the product of the maximum isometric force ($F_{M}^{0}$) and the muscle activation (*a*), force-velocity (*f*_*v*_), active force-length (*f*_*l*_) and passive force-length (*f*_*p*_) relationships, and tendon force is the product of the maximum isometric force and the tendon force-length (*f*_*t*_) relationship. In simulation PAS, passive force-length relationship ($f_{p_{N}}$) is updated based on the calibration of passive joint angle-moment relationships in the generic musculoskeletal model. In simulation TEN, the tendon force-length relationship ($f_{t_{N}}$) is updated based on personalized tendon stiffness values. In simulation EMG, the performance criterion incorporates a term to track experimental EMGs.

We computed muscle excitations, states, and state derivatives using four degrees of individualization in our simulations by (1) starting from the scaled generic model and muscle recruitment based on minimum effort, then consequently including (2) calibrated parameters of the muscle passive forces from the generic model, (3) subject-specific personalization of Achilles and quadriceps tendon stiffnesses, and (4) muscle controls informed with recorded EMGs (Fig 1). The following simulations were performed in order of increasing complexity:

GEN: Minimal muscle effort with generic passive force. Muscle excitations were computed assuming optimal motor control that minimizes the sum of squared muscle activations.

PAS: Minimal muscle effort with calibrated passive force. Muscle excitations were computed assuming optimal motor control that minimizes the sum of squared muscle activations.

TEN: Minimal muscle effort with calibrated passive force and personalized tendon stiffness. Muscle excitations were computed assuming optimal motor control that minimizes the sum of squared muscle activations. Personalized Achilles and quadriceps tendon stiffnesses are as in the simulation workflow EMG.

EMG: Muscle controls derived from minimal muscle effort and EMGs, with calibrated passive force and personalized tendon stiffness. Muscle excitations of the biceps femoris long head, semitendinosus, vastus lateralis and medialis, tibialis anterior, gastrocnemius lateralis and medialis, and soleus tracked the recorded time-series EMG data. The remaining muscle excitations were computed, assuming optimal motor control to minimize the sum of squared muscle activations. Thus, the objective function minimized two quantities: the sum of squared muscle activations and the deviation between estimated excitations and recorded EMGs (terms not equality weighted in the objective function). Achilles and quadriceps tendon stiffnesses were added as design variables and optimized using all the gait cycles simultaneously per subject.

In all simulations, inverse kinematics and inverse dynamics solutions were prescribed. Also, muscle excitations were estimated by minimizing activations squared while imposing that the moments produced by the muscle-tendon unit should equal the inverse dynamics joint moments. To guarantee the feasibility of the optimization problem, we added ideal non-physiological actuators at each joint, called reserve actuators. These actuators accounted for the joint moments that the muscle-tendon actuators could not reproduce, and their use was discouraged by penalizing their squared magnitude in the cost function. Detailed information about the formulation of the optimization problems: constraints, objective function’s weights, etc., is presented in S1 Appendix (section B: Simulation workflows).

#### 2.4.4 Metabolic energy models

Muscle metabolic rates were estimated using six metabolic energy models: Umberger et al. (UM03) [14], Bhargava et al. (BH04) [12], Houdijk et al. (HO06) [15], Lichtwark and Wilson (LW07) [16], Umberger (UM10) [17] and Uchida et al. (UC16) [38]. All were based on the first law of thermodynamics, and their main features are summarized in the supplementary material (S1 Table). Briefly, muscle excitations, states, and state derivatives over a gait cycle were used to compute the total rate of metabolic energy for each muscle based on its contractile element work rate and heat rate components (Eq 2):

$${\dot{E}}_{i}(t)={\dot{W}}_{CEi}(t)+{\dot{H}}_{Ai}(t)+{\dot{H}}_{Mi}(t)+{\dot{H}}_{SLi}(t)$$ (2)

where, for muscle *i* at time *t*, ${\dot{E}}_{i}$ is the metabolic rate, ${\dot{W}}_{CEi}$ is the contractile element work rate, ${\dot{H}}_{Ai}$ is the heat rate due to activation, ${\dot{H}}_{Mi}$ is the heat rate due to maintenance, and ${\dot{H}}_{SLi}$ is the heat rate due to shortening and lengthening. In all models, except LW07, heat rates depend on the muscle mass, computed based on muscle volume (see Musculoskeletal model, scaling method, and inverse kinematics and dynamics) and muscle density [14].

Eccentric muscle work could result in negative metabolic rate estimation, which would imply that muscles can absorb/gain energy during eccentric contractions. Although there is evidence that muscles can absorb heat during lengthening [39], it is physiologically questionable whether all chemical reactions are reversible when performing negative muscle work [40]. In this regard, in the LW07 model, half of the contractile element work is considered to be dissipated as heat and the other half as energy absorption/gain. UM10 only includes concentric contractile element work in estimating the metabolic rate, and UC16 constrains the metabolic rate to be non-negative. The HO06 model did not describe the heat rate for eccentric contraction; nonetheless, its heat rate is typically assumed as zero [24,25] which leads to negative metabolic rates at eccentric contractions. It is also possible to estimate non-negligible negative metabolic rates in the original formulations of the UM03 and BH04 models. As negative metabolic rate is not explicitly addressed in BH04 and HO06 models, and as the UM03 model was updated to UM10 in part for this reason, we opted to further modify ${\dot{H}}_{SLi}$ in BH04, HO06 and UM03 when ${\dot{E}}_{i}<0$ to assume that negative metabolic rate is dissipated by heat, as per Uchida et al. [18]; ${\dot{H}}_{SLi}$ in Eq 2 is replaced by ${\dot{H}}_{SLi,mod}$ for ${\dot{E}}_{i}<0$ (Eq 3):

$${\dot{H}}_{SLi,mod}(t)=-{\dot{W}}_{CEi}(t)-{\dot{H}}_{AMi}(t)\;for\;{\dot{E}}_{i}(t)<0$$ (3)

This modification prevents the metabolic rate in the BH04, HO06, and UM03 models from becoming negative.

**Fig 2:**
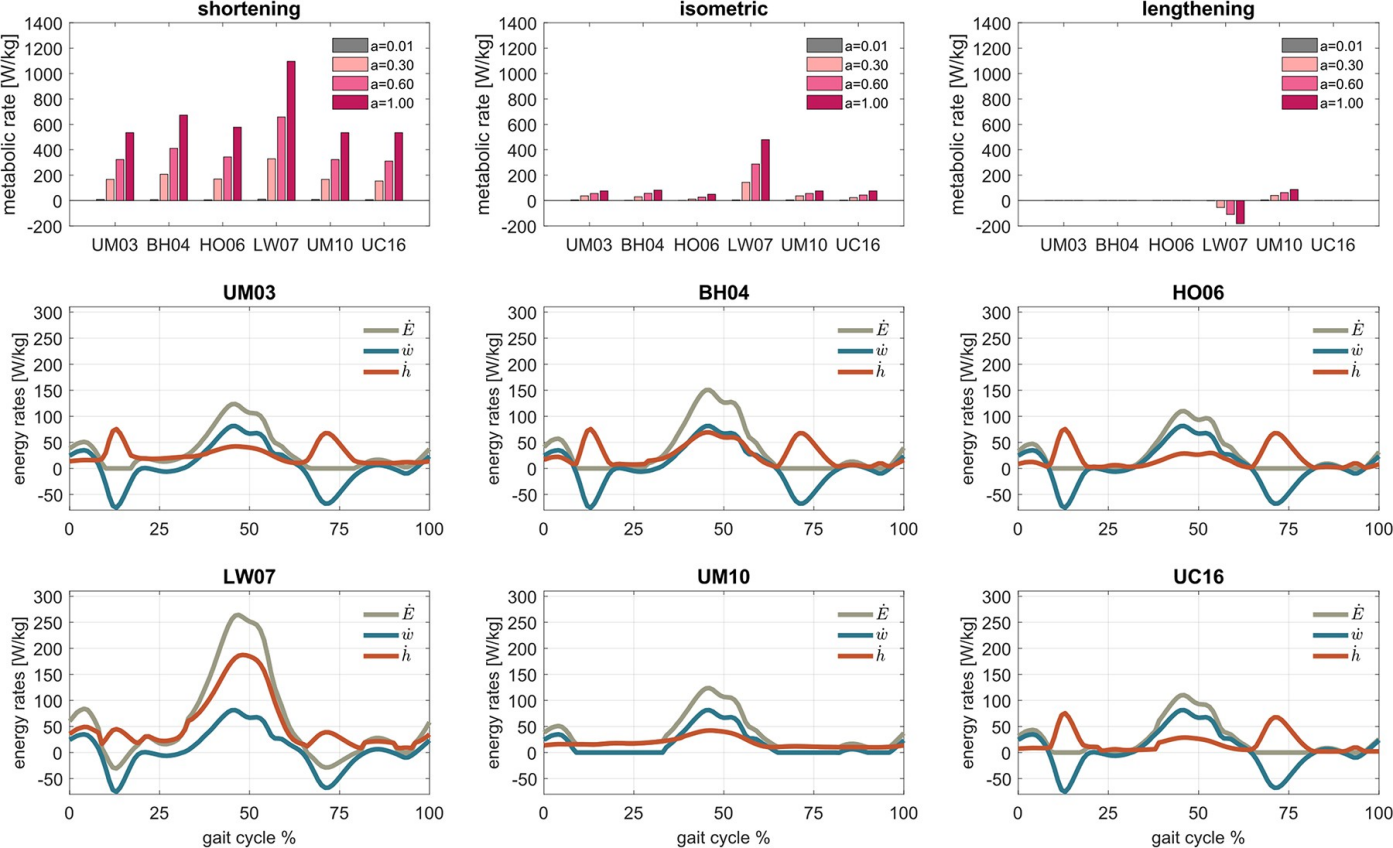
Metabolic rates in the soleus at different levels of muscle activations during shortening, isometric, and concentric contractions (first row), and metabolic ($\dot{E}$), work ($\dot{w}$), and heat ($\dot{h}$) rates during walking (second and thrid row). Metabolic rates were computed using six models: Umberger et al. [14] (UM03), Bhargava et al. [12] (BH04), Houdijk et al. [15] (HO06), Lichtwark and Wilson [16] (LW07), Umberger [17] (UM10), and Uchida et al. [18] (UC16). The ratio of slow twitch muscle fiber, muscle mass, optimal fiber length, and maximum voluntary contraction is assumed as 0.8, 0.48 kg, 4.5 cm, and 10 [optimal fiber lengths/second], respectively. Simulated muscle metabolic rate as computed from one representative subject walking at preferred speed.

To illustrate the influence of muscle states on metabolic rates as well as the differences between models, the metabolic energy rates of the soleus at various levels of muscle activations and muscle states (Fig 2), as well as metabolic energy rates and muscle efficiency across fiber lengths and fiber velocities (S2 Fig) were computed. During shortening contractions, the UM03 and UM10 models have identical shortening metabolic rates, and their energy efficiency varies greatly within low activation levels. The LW07 model predicted the highest metabolic rates, followed by the BH04, HO06, and UC16 models. The energy efficiency of the BH04, HO06, and UC16 models is practically invariant across activation levels, while the energy efficiency of the LW07 model is constant across activation levels. During isometric contractions, the LW07 predicted the highest metabolic rate, followed by the BH04 model and then the UM03 and UM10 models, which have identical metabolic rates. A somewhat lower value is predicted by the UC16 model and then the HO06 model. All models predict no metabolic rates during lengthening contractions except the UM10 and LW07 models, where the latter predicted net negative energy.

#### 2.4.5. Data and statistical analysis

We evaluated the agreement between computed muscle excitations and muscle fiber lengths from each simulation workflow and available experimental observations. Specifically, we compared the computed muscle excitations of most muscles in the musculoskeletal model with digitalized values from Perry’s reported fine-wire EMG observations. [41]. We also compared the computed normalized fiber lengths of gastrocnemius lateralis and medialis, soleus, and vastus lateralis with reported values from ultrasound studies across walking speeds [42–47]. We then identified the workflow with the highest accuracy, i.e., whose computed muscle-tendon dynamics agreed overall best with experimental findings. For this purpose, we computed the correlation coefficient and root-mean-square error (rmse) between computed and experimental muscle excitations and fiber lengths.

We evaluated metabolic energy models by comparing the experimental whole-body average metabolic rates obtained from spiroergonometry and estimated whole-body average metabolic rates from each simulation workflow. The experimental whole-body average metabolic rate was computed based on the representative (3 minutes averaged) oxygen and carbon dioxide rate values for each speed using the Brockway equation [4]. The estimated whole-body average metabolic rate was computed based on the metabolic rates from metabolic energy models, plus a basal rate assumed to be 1.2 W/kg [14]. The estimated whole-body average was calculated as the integral over time of the time-series metabolic rates of muscles divided by the duration of the gait cycle and multiplied by two to account for two legs (as we simulated the dynamics of one leg).

We evaluated the time-series metabolic rates in one leg computed from metabolic energy models. Specifically, we compared metabolic rates in workflows with increasing complexity, i.e., between workflow GEN and PAS, PAS and TEN, and TEN and EMG, at each time instance using a one-dimensional statistical non-parametric mapping toolbox (SnPM, *α* = 0.05, two-tailed paired t-test) [48]. Discrete variables, specifically timing of local maxima and minima, were compared with the Wilcoxon signed-rank test.

The relative costs of the stance (initial foot contact to toe-off) and swing (toe-off to next foot contact) phases were computed, normalized by the total energy cost of the gait cycle. The estimated metabolic rates of the muscle function groups at the ankle, knee, and hip joints were computed as a sum contribution of the uniarticular and biarticular muscles at each joint. For biarticular muscles, the metabolic rate was distributed between joints based on the ratio of moment arms, as implemented by Uchida et al. [18].

We used a repeated measure correlation [49] to determine whether correlations exist between estimated and experimental whole-body metabolic rates, between the estimated relative cost of the muscle groups and walking speed, and between the estimated relative cost of the stance and swing phases and walking speed. Repeated measure correlation, proposed by Bland and Altman, was chosen as it is suitable for establishing linear relationships between predicted and experimental metrics adjusting for inter-subject variability [50]. Linear regression, including all subjects and walking trials, would be unsuitable as it violates the assumption of independence of observations [49]. Repeated measure correlation determines a common within-subjects association, i.e., regression slope, and removes measured variance between subjects. We also reported the differences/errors between estimated and experimental whole-body average metabolic rates. Finally, we verified that the marker trajectory errors are within recommended values from Hicks et al. [26] and that the computed joint angle and moment (inputs in the simulation workflow) are in accordance with prior studies in the literature. Detailed information about the marker trajectory errors, joint angle, and joint moments are presented in S1 Appendix (section D: Simulation verification).

## 3. Results

### 3.1. Evaluation of muscle excitations, fiber lengths, and tendon stiffness estimations

**Fig 3:**
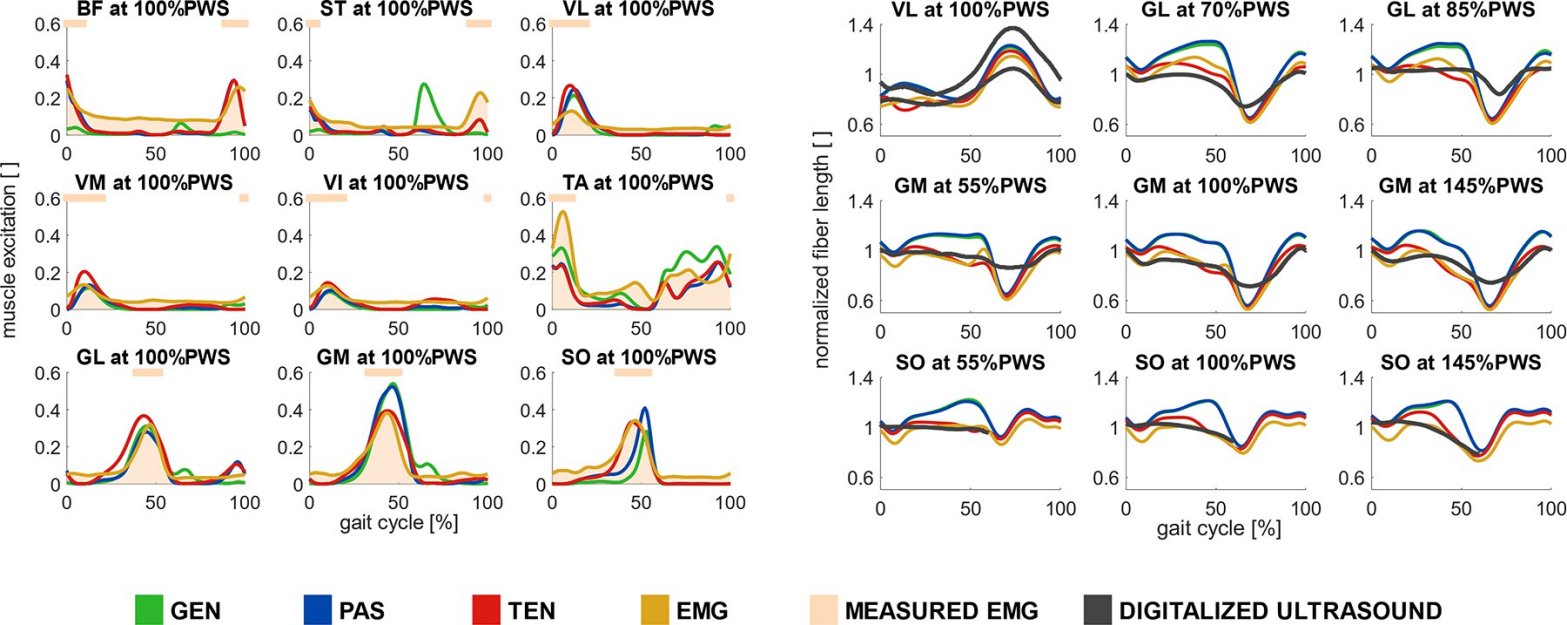
*Computed and experimental muscle excitations and fiber lengths*. Muscle excitations of biceps femoris long head (BF), semitendinosus (ST), vastus lateralis (VL), vastus medialis (VM), tibialis anterior (TA), gastrocnemius lateralis (GL), gastrocnemius medialis (GM) and soleus (SO) during the gait cycle at preferred walking speed (PWS) (left) and normalized fiber lengths of vastus lateralis, gastrocnemius lateralis, gastrocnemius medialis, and soleus during the gait cycle across walking speeds with four simulation workflows: Minimal muscle effort with generic passive force (GEN), with calibrated passive force (PAS), with calibrated passive force and personalized tendon stiffness (TEN), and EMG-informed with calibrated passive force and personalized tendon stiffness (EMG). Simulated muscle excitations and normalized fiber lengths represent the average values among all subjects. Measured EMGs represented the average values among all subjects and were scaled using optimization variables in EMG. Experimental values of fiber lengths were obtained by digitalizing previously reported experimental findings using ultrasound in vastus lateralis [42,43], gastrocnemius lateralis [44,46], gastrocnemius medialis [45] and soleus [47]. Experimental fiber lengths were normalized based on average values reported from a muscle architecture data set [52] when not otherwise reported in experimental studies. Horizontal lines above muscle excitations indicate on/off timing for EMG signals, defined as >50% excitation.

Calibration of passive forces improved the estimation of muscle excitations of the muscle function group at the hip and knee joints compared to the generic simulation workflow, and the EMG-informed simulation workflow estimated large excitations in small muscles. Salient muscle excitation features of adductor longus, extensor digitorum longus, extensor hallucis longus, flexor digitorum longus, flexor hallucis longus, gastrocnemius medialis and lateralis, gluteus medialis, gracilis, peroneus brevis and longus, sartorius, soleus, tibialis anterior, tibialis posterior, vastus intermedius, lateralis, and medialis, were estimated with all simulation workflows (Figs 3 and S3). Excitations of the muscle function groups at the hip and knee joints–adductor magnus, bicep femoris long head, gluteus maximus, semimembranosus, and semitendinosus–were better modelled in the simulations with calibrated passive forces (PAS, TEN, and EMG), compared to simulations with generic passive forces (Figs 3 and S3). The salient feature of the rectus femoris excitation during the swing phase was better modelled with calibrated passive forces (PAS, TEN, and EMG), while the salient features of adductor brevis, iliacus, psoas excitations were not well estimated in any of the simulation workflows (S3 Fig). The EMG-informed simulation workflow (EMG) estimated a similar on/off pattern than the simulation workflow TEN but estimated excitation substantially higher magnitudes, especially for the rectus femoris and for small muscles around the ankle (e.g., flexor digitorum longus, S3 Fig).

**Fig 4:**
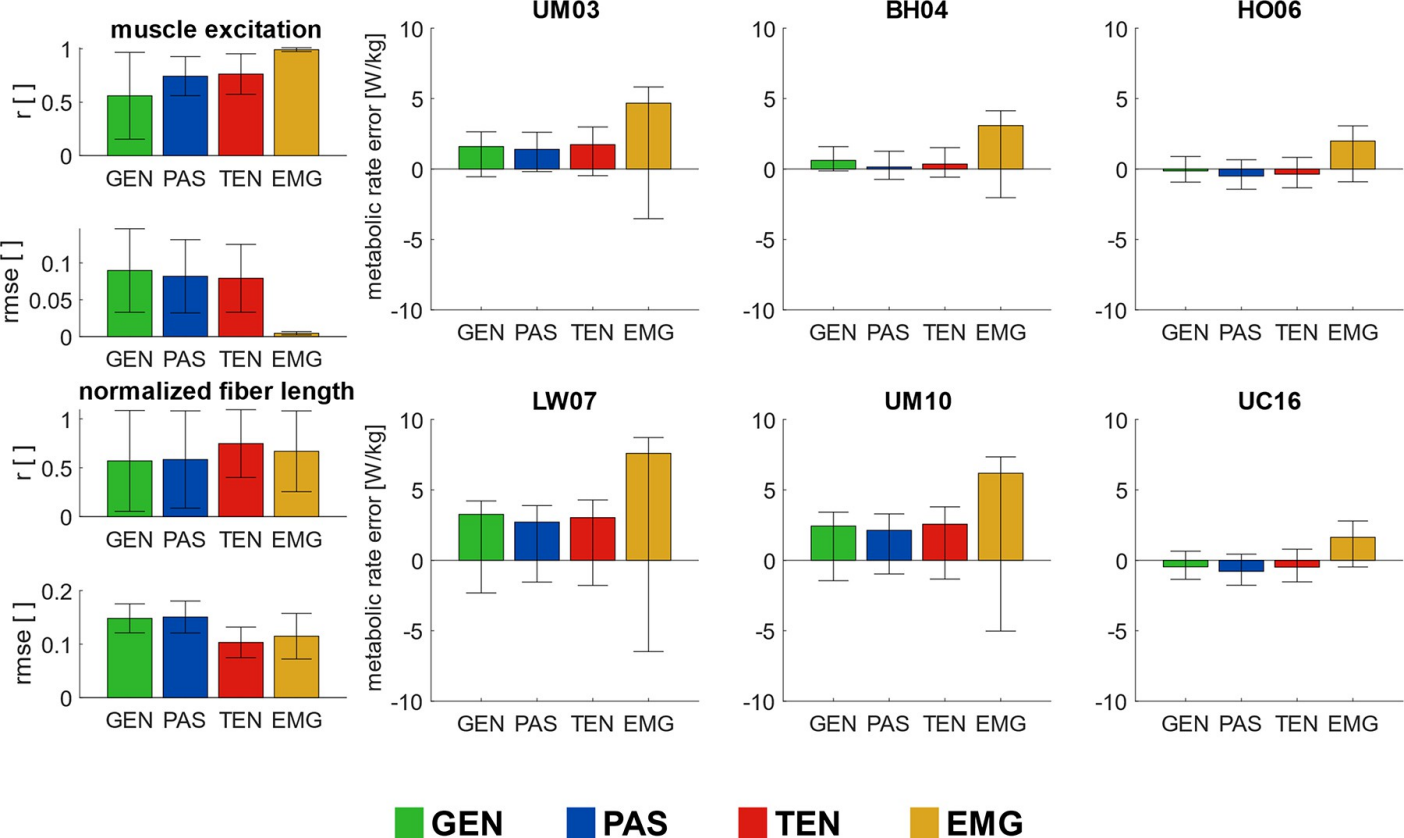
*Quantitative differences between computed and experimental muscle excitations*, *fiber lengths*, *and whole-body average metabolic rates*. Correlation coefficient *r* and rmse (column 1) between computed and experimental muscle excitations and fiber lengths, and differences between estimated and experimental metabolic whole-body average metabolic rates (columns 2–4, W/kg) with four simulation workflows: Minimal muscle effort with generic passive force (GEN), with calibrated passive force (PAS), with calibrated passive force and personalized tendon stiffness (TEN), and EMG-informed with calibrated passive force and personalized tendon stiffness (EMG), using six metabolic energy models: Umberger et al. [14] (UM03), Bhargava et al. [12] (BH04), Houdijk et al. [15] (HO06), Lichtwark and Wilson [16] (LW07), Umberger [17] (UM10), and Uchida et al. [18] (UC16). Bar plots shown mean value and one standard deviation computed among all subjects and walking trials.

Personalization of tendon stiffness improved the estimation of the normalized fiber lengths compared to the generic tendon stiffness values, and the computed fiber lengths were affected by informing the muscle control with EMGs. The personalized Achilles tendon stiffness (~160 N/mm) was within the *in vivo* measured values (141–170 N/mm) by Stenroth et al. [51] (see S1 Appendix, section B: Simulation workflow). Also, the personalized quadriceps tendon estimated nearly isometric contraction of vastus lateralis during loading response with the simulation workflow EMG, which aligns with previous experimental observations [42,43]. Simulation workflows without tendon stiffness personalization failed to estimate the operating range of plantarflexors as suggested in the literature, i.e., models estimated that plantarflexor muscles operated at the ascending limb or plateau (0.75 < normalized fiber length < 1.05) instead of the descending limb (normalized fiber length > 1.05) (Fig 3). Fiber lengths were not sensitive to the calibration of passive force-length relationship; muscles worked at the same operating range between the simulation workflows GEN and PAS. However, fiber lengths were affected by informing muscle excitations with EMGs. The simulation workflows TEN and EMG estimated somewhat different fiber lengths, though they operated in similar regions. The simulated vastus lateralis contracted concentrically and then eccentrically during the loading response in the simulation workflow TEN. Overall, the simulation workflow TEN showed the best estimation of muscle excitations and fiber lengths among all the simulation workflows (Fig 4).

### 3.2. Evaluation of whole-body average metabolic rate estimations

**Fig 5:**
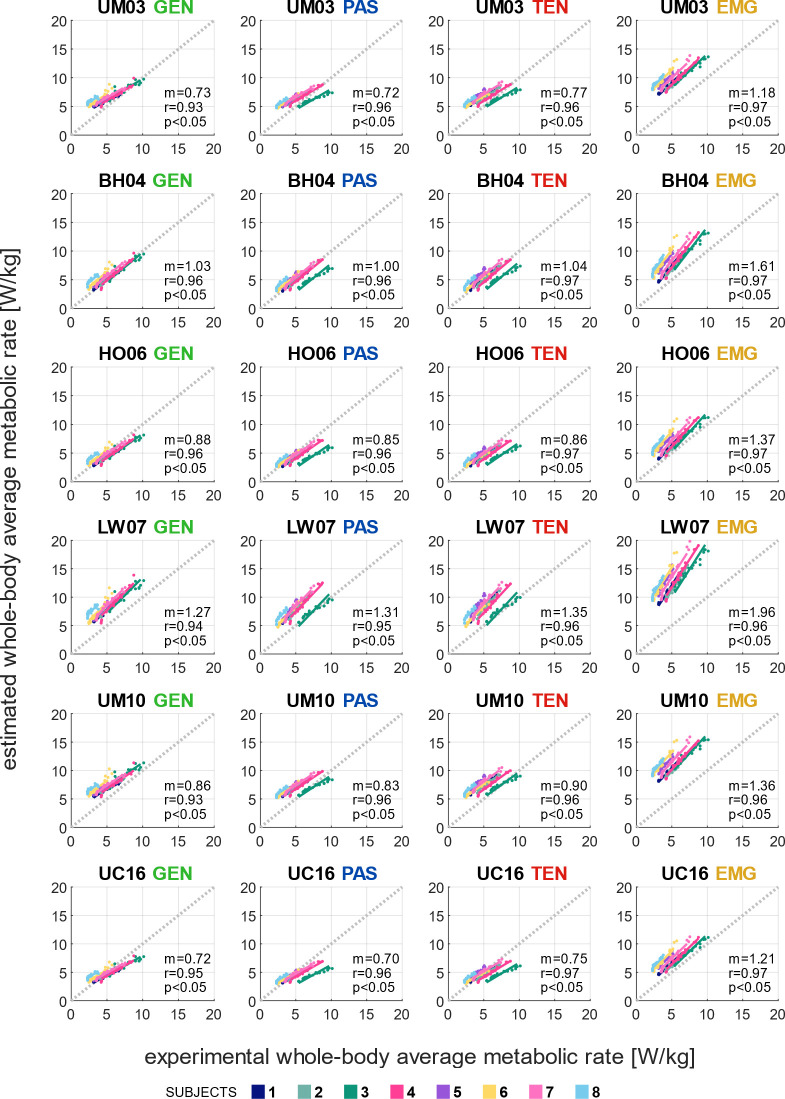
Repeated measures correlation between estimated and experimental whole-body average metabolic rates. Repeated measures correlation between estimated and experimental whole-body average metabolic rate [W/kg] over a range of metabolic demand with four simulation workflows: Minimal muscle effort with generic passive force (GEN), with calibrated passive force (PAS), with calibrated passive force and personalized tendon stiffness (TEN), and EMG-informed with calibrated passive force and personalized tendon stiffness (EMG), using six metabolic energy models: Umberger et al. [14] (UM03), Bhargava et al. [12] (BH04), Houdijk et al. [15] (HO06), Lichtwark and Wilson [16] (LW07), Umberger [17] (UM10), and Uchida et al. [18] (UC16). Each color represents an individual subject. An ideal relationship, equivalent to y = x is illustrated as a dotted line. For each simulation workflow and metabolic energy model, the slope m, correlation coefficient r, and p-value of the null hypothesis that no correlation between estimated and experimental metabolic rate exists are shown as means of all subjects.

Simulating muscle dynamics with calibrated muscle passive forces and personalization of tendon stiffness did not substantially improve the whole-body average metabolic rate estimation compared to the generic simulation workflow, and the EMG-informed simulation workflow overestimated metabolic rates. The differences and a linear relationship between the estimated and experimental whole-body average metabolic rate, with slope m and correlation coefficient r for all models and workflows, were analyzed for the full range of walking speeds (Figs 4 and 5). The lowest differences compared to experimental metabolic rates were observed in the simulations workflows GEN, PAS, and TEN with the BH04, HO06, and UC16 models (Fig 4). The estimated whole-body average metabolic rate correlated well with the experimental measurements with the generic simulation workflow (r≥0.93) (GEN); and with the inclusion of calibrated passive forces and personalized tendon stiffness (TEN), the correlation slightly increased (r>0.96) for all metabolic energy models (Fig 5). The correlation slopes were closest to 1.0 with the BH04 model (m = 1.04), followed by the UM10 and HO06 models (m = 0.90 and 0.86, respectively). Including EMGs in the muscle control increased the estimated metabolic rate, led to overestimating the correlation slopes, and did not improve the correlation coefficient in most metabolic energy models. Overall, the LW07 provided the lowest agreement between estimated and experimental metabolic rates among the simulation workflows, while the BH04 model showed the highest.

### 3.3. Estimated metabolic rates among levels of individualization

**Fig 6:**
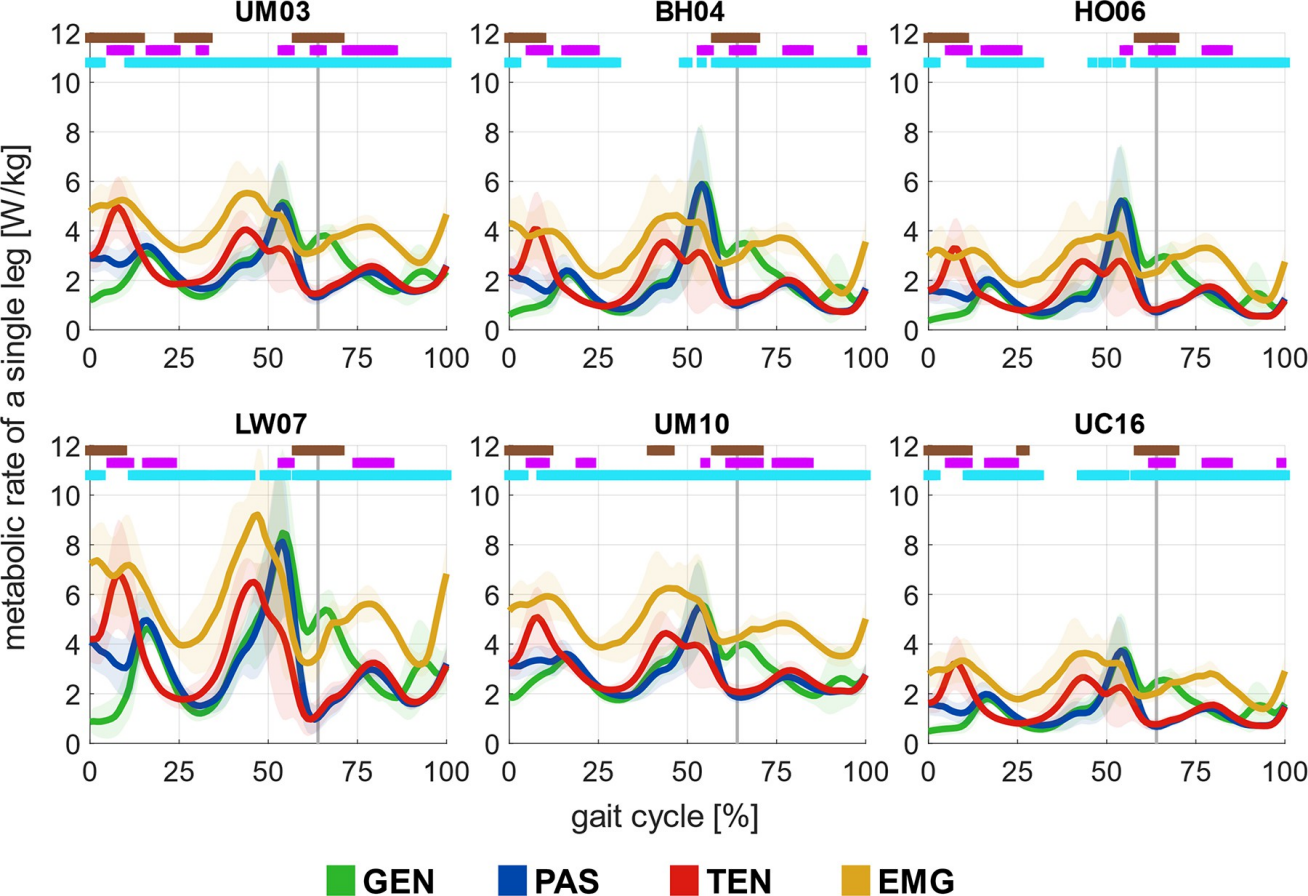
*Estimated metabolic rates among the simulation workflows*. Estimation of metabolic rates in one leg at preferred walking speeds with four simulation workflows: Minimal muscle effort with generic passive force (GEN), with calibrated passive force (PAS), with calibrated passive force and personalized tendon stiffness (TEN), and EMG-informed with calibrated passive force and personalized tendon stiffness (EMG), using six metabolic energy models: Umberger et al. [14] (UM03), Bhargava et al. [12] (BH04), Houdijk et al. [15] (HO06), Lichtwark and Wilson [16] (LW07), Umberger [17] (UM10), and Uchida et al. [18] (UC16). The estimated metabolic rate represented the average values among all subjects, scaled by their mass. Statistically significant differences (SnPM test, p<0.05) are shown in horizontal lines above each figure. They indicate differences between workflows GEN and PAS (brown), between workflows PAS and TEN (purple), and between workflows TEN and EMG (cyan). The vertical line represented the toe-off event.

While within a given simulation workflow, the estimated magnitude, timing, and duration of metabolic energy peaks were metabolic model-dependent (Fig 6), the levels of individualization in the various workflows influenced the estimated metabolic rates time-series. At preferred walking speed, the generic simulation workflow (GEN) estimated metabolic rate peaks near mid-stance, pre-swing, and initial swing, largely associated with action of the knee extensors, plantarflexors, and hip flexors, respectively. With calibrated passive forces (PAS), the overall metabolic rates are much lower during early swing (i.e., 0 to ~10% of the gait cycle, p < 0.05 (all p values in this section are according to the SnPM test)) and higher in early stance (i.e., ~58 to 69% of the gait cycle, p < 0.05) coinciding with higher computed excitation of hip extensors and hip adductors in early stance and lower hip flexor excitation in early swing (Fig 3) than with the workflow GEN. With personalized tendon stiffness (TEN), compared to both workflow GEN and PAS, the peak metabolic rates during pre-swing tend to be lower and earlier, and over a longer duration than with workflow PAS. The peak metabolic rate with workflow TEN was lower than with workflow PAS in most of the metabolic energy models (~55% of the gait cycle, p < 0.05 in all models except UC16), associated with lower but earlier plantarflexor excitations near pre-swing. Also, with workflow TEN, the peak near early stance was higher than with the workflow PAS (~8% of the gait cycle, p < 0.05), associated with higher excitation of the vastus lateralis and medialis in early stance. With further information from EMGs (EMG), the estimated metabolic rate again showed similar timing of metabolic rate peaks compared with workflow TEN, but with a noticeably high offset throughout most of the gait cycle (p < 0.05), associated with overall higher computed activations in small ankle and knee muscles that were not tracked by EMG signals (S3 Fig).

### 3.4. Estimation of the metabolic rates at the whole-body and muscle function group level

**Fig 7:**
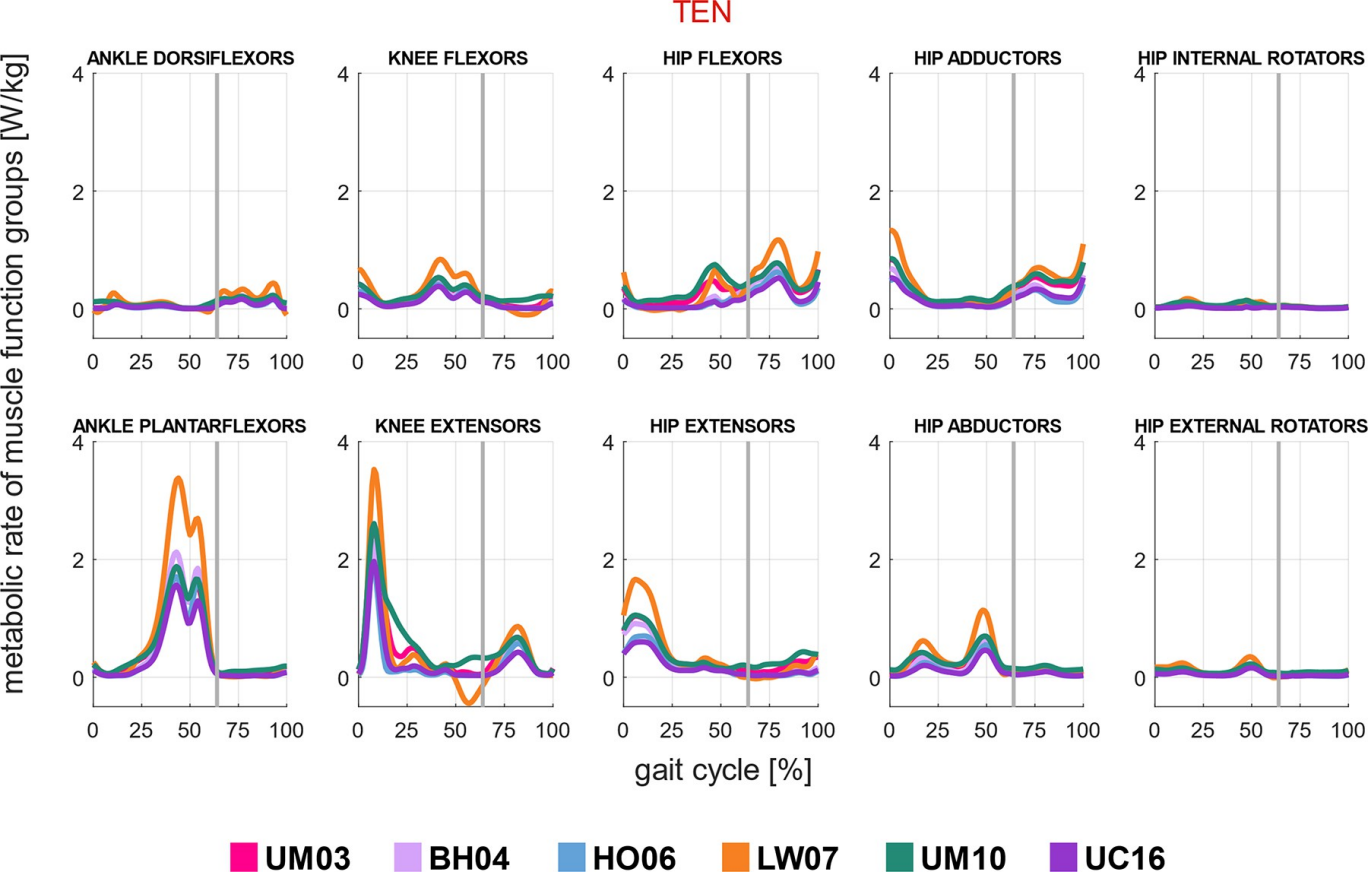
*Estimated metabolic rates of muscle function groups*. Estimation of metabolic rates in the muscle function groups [W/kg] at preferred walking speed with the simulation workflow based on minimal muscle effort with calibrated passive force and personalized tendon stiffness (TEN), using six metabolic energy models: Umberger et al. [14] (UM03), Bhargava et al. [12] (BH04), Houdijk et al. [15] (HO06), Lichtwark and Wilson (LW07) [16], Umberger [17] (UM10), and Uchida et al. [18] (UC16). The estimated metabolic rates represented the average values among all subjects, scaled by their mass. The vertical line represented the toe-off event.

Metabolic peak rates were more pronounced as walking increased. At the muscle function group level, the simulation workflow with calibrated muscle passive forces and personalization of tendon stiffness (TEN) estimated that the first peak was mainly caused by knee and hip extensors; the second peak by plantarflexors and abductors; and the third by hip flexors, hip adductors, and knee extensors at preferred walking speed (Fig 7). In all the metabolic energy models, except with the UM10 model, the highest cost is accounted by the plantarflexors (18.4% to 24.7%), followed by the knee extensors (14.7% to 18.1%) and hip extensors (12.2% to 13%) (S2 Table). The hip adductors and abductors combined accounted for approximately 20% of the overall energy cost, and hip internal and external rotators required almost negligible cost. Detailed information about the comparison between metabolic rates is presented in S1 Appendix (section C: Comparison of metabolic estimations among simulation workflows).

**Fig 8:**
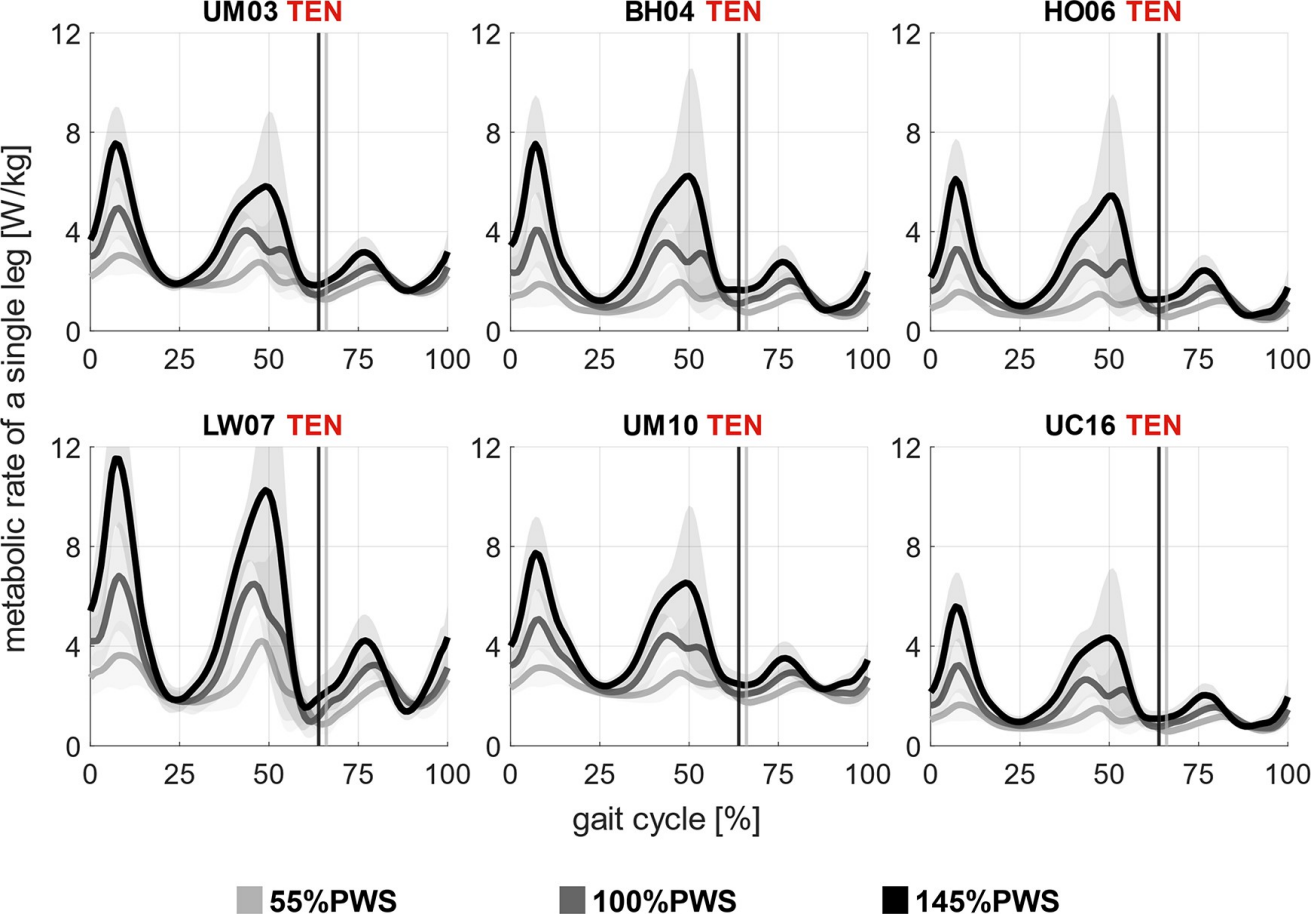
*Estimated whole-body metabolic rates across walking speeds*. Estimation of the whole-body metabolic rates [W/kg] at 55% PWS (light grey), 100% PWS (dark grey), and 145% PWS (black) with the simulation workflow based on minimal muscle effort with calibrated passive force and personalized tendon stiffness (TEN), using six metabolic energy models: Umberger et al. [14] (UM03), Bhargava et al. [12] (BH04), Houdijk et al. [15] (HO06), Lichtwark and Wilson [16] (LW07), Umberger [17] (UM10), and Uchida et al. [18] (UC16). The estimated metabolic rates represented the average values among all subjects, scaled by their mass. The vertical line represented the toe-off event across walking speeds.

Walking speed influenced the estimated metabolic rates throughout the gait cycle primarily by modulating the metabolic rate peaks as the speed increased. At the highest speeds, the peak near pre-swing was dominant, and at slow walking speeds, the peaks were broadly similar, wherein the peak near the early stance was lower than the others (Fig 8). Also, in all the metabolic energy models, we found a low to moderate correlation between the relative cost of the muscle function group (with respect to the total energy cost of the gait cycle) and the walking speeds (S4 Fig). In all the models, the relative cost of the ankle plantarflexors substantially increased with walking speed (p<0.05), while hip flexors, adductors, and abductors decreased (p<0.05). Trends in other muscle function groups were less pronounced (or not significant) and dependent on the metabolic energy model.

### 3.5. Estimation of the relative costs of gait phases

**Fig 9:**
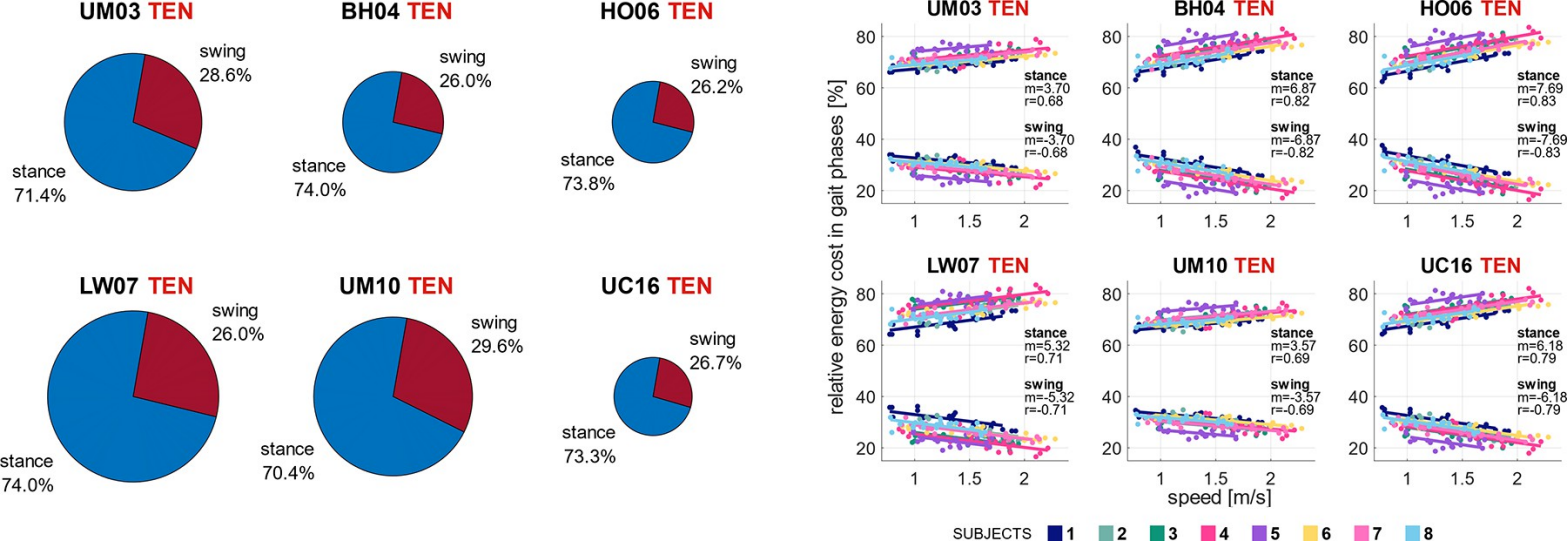
*Estimated relative cost of gait phases*. Cost of the stance and swing phase relative to the total energy cost in a gait cycle during preferred walking speed [left] and vs. walking speeds [right] (in percentage) with the simulation workflow based on minimal muscle effort with calibrated passive force and personalized tendon stiffness (TEN), using six metabolic energy models: Umberger et al. [14] (UM03), Bhargava et al. [12] (BH04), Houdijk et al. [15] (HO06), Lichtwark and Wilson [16] (LW07), Umberger [17] (UM10), and Uchida et al. [18] (UC16). Pie chart areas are scaled based on the total energy cost. Individual subjects are illustrated in different colors, and the slope and correlation coefficient from repeated measures correlation is indicated. The P-value was <0.05 for all correlations.

The metabolic energy models in the simulation framework with the highest accuracy estimated that the relative cost of the swing phase accounted for slightly more than one-quarter of the total energy cost of the gait cycle and decreased with faster walking speed. At the preferred walking speed, the simulation workflow with calibrated passive forces and personalization of tendon stiffness (TEN) estimated a similar relative cost of the swing phase among metabolic energy models. It varied from 26% with the BH04 model to 29.6% with the UM10 model (Fig 9). In addition, we found a significant (p<0.05) yet moderate correlation between the relative cost of gait phases and walking speed in all the metabolic energy models (Fig 9). The correlation slope magnitudes varied from 3.57 with the UM10 model to 7.69 with the HO06 model.

## 4. Discussion

Models with calibrated passive forces and personalized tendon stiffness better estimate muscle excitations, fiber lengths, and whole-body average metabolic rates across walking speeds compared to generic simulation workflows. Perhaps unexpectedly, models that estimate muscle excitation with information from experimentally-measured EMGs did not estimate metabolic rate accurately. This observation can be attributed to experimental limitations that make it possible to constrain only a subset of muscle EMGs. Muscle excitations are commonly validated by their agreement with EMGs [26]; muscle excitations informed by recorded EMGs should be more reliable than muscle excitations obtained from only optimization routines. By tracking EMGs closely (+/-0.01 tracking bound), our simulation has a relatively low sensitivity to the objective function’s weights. Yet, estimated excitations in most of the non-tracked muscles at the ankle and knee increased. We evaluated whether we could avoid this increment by tracking EMGs less closely (+/-0.05 tracking deviation), but found instead estimations in which the muscle-tendon states depended on the weights of the objective function, which is likely not generalizable. We simultaneously tracked recorded EMGs and minimized muscle activations, which derived into opposing goals in the objective function; see further discussion in S1 Appendix (section B: Simulation workflows). Another alternative was to include only muscles whose recorded EMGs were available, also called EMG-driven simulations in literature [53]. This approach also poses a challenge as EMGs from deep muscles cannot be recorded using surface electromyography [23]; thus, the degree to which deep muscles influence the estimations is uncertain. Furthermore, higher estimated muscle excitations led to overestimated metabolic rates in the simulation workflow EMG compared to TEN. This finding suggests that informing muscle control with EMGs in only a subset of muscles might propagate modelling errors that worsen estimates of muscle excitations and metabolic rate. Future work using EMG-informed simulations might benefit by adjusting temporal parameters to models of EMGs-to-force production better or by finding a better suitable trade-off between tracking EMGs and minimizing muscle effort. We determined that simulation workflow TEN had the highest accuracy in estimating muscle activations and fiber lengths among the simulation workflows and, therefore, used it for subsequent muscle metabolic rate analyses.

Accounting for the individualization of muscle passive force-length relationship resulted in higher accuracy in the estimated muscle control. The computed muscle control trajectory is a result of the muscle-tendon properties and the performance criterion. We observed that simulating muscle dynamics with the same objective function using calibrated muscle passive force-length relationships resulted in more accurate muscle excitations, most notably at the knee flexors, than using generic values. The original model proposed by Rajagopal et al. included the latest dataset of muscle architecture but no information about the joint passive moments [31]; our findings suggest that joint passive moments are critical for accurately estimating muscle excitations around the knee and hip. Likely, other musculoskeletal models derived from subject-specific data [54] might also benefit from incorporating a description of the joint passive moments.

Compared to generic values, the calibrated values tended to produce passive forces at relatively longer normalized fiber lengths and with less stiffness. The passive moment-angle relationships at the ankle, knee, and hip tended to be more compliant, which in turn affected the computed muscle activity. For instance, with calibrated passive plantarflexion moments, which tended to be more compliant than the generic passive moments, more soleus activation was required to plantarflex the foot during mid- and terminal stance; conversely, with stiffer passive moments, less synergistic muscle activity was required. Likewise, with calibrated moments, nearly zero passive moments are produced at small knee flexion and different hip flexion angles; muscle activation must thus generate practically the entire moment in positions near the neutral angle. This mechanism explains why muscles such as in the biceps femoris long head are active during the beginning and the end of the gait cycle with calibrated passive forces but not with generic passive forces.

Modelling the Achilles tendon as highly compliant, with stiffness values within the measured values reported by Stenroth et al. [51], resulted in higher fiber length agreement with ultrasound imaging and lower metabolic energy peaks than with a stiff tendon. Identifying muscle-tendon properties using EMGs requires motion data from multiple trials to ensure it represents subject-specific characteristics [55]. These findings suggest that our method might be sufficient to capture this property in healthy individuals. Also, it showed the importance of modeling tendon compliance in the energetic aspect of locomotion. Tendon compliance is associated with high energy efficiency during walking and running [16,18]. In a previous simulation study, higher Achilles tendon compliance resulted in lower metabolic rates due to lower contractile element work and nearly isometric contraction during mid- and terminal stance, compared to a stiffer generic stiffness [16]. The Achilles tendon stores energy during these gait phases to later support plantarflexion motion, thus behaving as a “catapult” to assist the push-off action in pre-swing [56]. We estimated that the energetic cost associated with this mechanism resulted in an earlier peak metabolic rate (48% gait cycle with TEN vs. 55% with PAS, Wilcoxon test, p<0.05 in 4/6 and p = 0.0625 in 2/6 metabolic energy models) and a lower peak metabolic rate during the push-off action in pre-swing (SnPM test, p < 0.05). A stiff Achilles tendon, related to morphological pathologies [57], does not exhibit this trade-off, which emphasizes the importance of modelling muscle-tendon mechanics with compliant tendons during walking if metabolic rates are an outcome of interest.

Modelling the quadriceps tendon as highly compliant showed somewhat the opposite effect than in the Achilles tendon. Interestingly, we observed that the metabolic peak rate during early stance increased, followed by a small decrease in energy cost compared to a generic stiffer quadriceps tendon. The fiber excursion of the vastus lateralis was larger than experimentally observed [42,43], which might have increased the positive muscle work rate and, therefore, overestimated the metabolic rate in the early stance. The main factor that might have obscured our estimation is the simplifications of the muscle-tendon lengths of the knee extensors. The quadriceps tendon and patellar ligament are lumped together as one parameter in the model we used. Yet, these passive structures differ in function and mechanical properties [58]. Including the patellar ligament would decrease the quadriceps tendon length and, thus, the estimated tendon compliance. A stiffer tendon implies its elongation is less sensitive to active force production [11], decreasing muscle fiber excursion. Future studies might consider incorporating the patellar ligament to better estimate the muscle fiber and metabolic rates at the knee extensor muscle group.

Our reported estimation of metabolic rates that are more accurate than prior comparison studies is likely attributable in part to the consideration of negative metabolic rates and to the complexity of the musculoskeletal model. It is not physiologically plausible to estimate net energy absorption, i.e., gaining energy through an eccentric muscle contraction, as also pointed out by Uchida et al. [18]. Miller observed that various metabolic energy models better estimated measured energy cost when eccentric (negative) work was removed from the metabolic cost computations [24]. We likewise truncated any non-negative metabolic rates. Our results disagreed somewhat with those of Koelewijn et al. [25], who found that metabolic energy costs with the BH04, HO06, UM03, and LW07 were underestimated, especially at high metabolic demands. Arguably, that we truncated the negative metabolic rate accounts for some of this difference, but the disagreement is also likely attributable to their omission of modelled muscles in the frontal plane. We estimated that nearly 1/5 of the total cost during walking is produced by the hip abductors and adductors.

Metabolic energy models estimated metabolic rates that correlated well with measured whole-body average metabolic rates, even in the generic simulation workflow, which might tempt dubious conclusions about time-series metabolic rate. Our generic simulation workflow (GEN) using the BH04 or HO06 models estimated an almost perfect agreement with measured whole-body average metabolic rates across walking speeds, even though this simulation workflow poorly estimated excitation and fiber lengths as compared to the simulation workflow TEN. Furthermore, the generic simulation workflow (GEN) estimated a large muscle energy cost in pre-and initial swing as a product of poor modelling of passive forces, estimated a high relative muscle energy cost in the swing phase with the BH04 or HO06 models (36.5 to 36.7%, respectively), and failed to capture attenuated metabolic rate peaks due to highly compliant tendons. For further discussion, see S1 Appendix (section C: Comparison of metabolic estimations among simulation workflows). In this regard, caution should be taken to interpret metabolic rates if the muscle activations and fiber lengths have not been validated.

Estimated metabolic rates of the muscle function groups with the BH04 and UM10 models are aligned with and extend findings from previous simulation studies. Umberger estimated that hip extensors, knee extensors, and ankle plantarflexors contributed the most to energy cost during the stance phase at a preferred walking speed [17], similar to our findings. Umberger also found that the most prominent metabolic rate peak occurred in loading response and was primarily attributed to hip extensors and hip extensors, wherein hip extensors accounted for the highest metabolic cost among muscle groups. We observed a similar timing of the metabolic rate peaks, though plantarflexors accounted for the highest metabolic cost in our simulations. In addition, we identified a metabolic rate peak during the initial swing caused by hip flexors, hip adductors, and knee extensors. In contrast, Umberger observed little to no contribution to metabolic rate from the knee extensors, particularly the rectus femoris. Discrepancies in metabolic rates are likely to be attributed to a higher muscle activation of the hip and knee extensors at heel strike compared to our simulation and the lack of rectus femoris activation during the initial swing estimated by Umberger. We estimated a rectus femoris activation peak at the initial swing, which is consistent with experimental observation [31,59]. Interestingly, Umberger estimated that the relative cost of the swing phase is 29%, which is nearly identical to our estimations for the UM10 model. Mohammadzadeh et al., using the UM10 model with modifications of Uchida et al. [18], estimated that plantarflexors accounted for 57% of the total metabolic cost, the highest among muscle function groups, followed by knee and hip extensors with 22% and 11%, respectively, and the ankle dorsiflexors and knee flexors, combined, with only 10% during walking at 1 m/s [60]. While we also identified that, among muscle function groups, the plantarflexors demanded the highest metabolic cost, even at low speeds, our estimated costs are approximately 1/3 of their reported magnitudes. The muscle actuators’ low fidelity to reproducing recorded knee and hip flexor joint moments might explain the discrepancy. Their study simulated walking with muscle-tendon actuators driven by recorded EMGs and optimized fiber and tendon lengths, wherein knee and hip flexors generated little to no moment compared to the inverse dynamics solution. Neglecting such muscle groups resulted in overestimated metabolic cost of ankle plantarflexors and knee extensors and failure to capture the metabolic cost of the hip flexors, the primary muscle function group for the swing phase according to our estimations. Similarly, Markowitz and Herr simulated walking using muscle-tendon actuators driven by recorded and digitalized EMGs and optimized muscle-tendon parameters to estimate muscle forces and metabolic rate using the UM10 model [27]. They estimated that the swing phase accounted for 26% of the total cost of walking, identical to our estimations with the BH04 model and slightly lower than the UM10 model (29.6%). In addition, we estimated a substantial cost of the hip adductor and abductors, nearly 20%, which was not described in the previous studies as they simulated hip articulation in the sagittal plane only. These comparisons suggest that estimated metabolic rates in muscle function groups might be obscured by the accuracy of modeled muscle-tendon mechanics. Future experimental studies might bring more insights to refine the estimates of metabolic cost.

The relative energy cost of the gait phases is related to fundamental principles of walking dynamics. An inverted pendulum model is frequently used to describe the dynamics of the leg during the stance phase—a high degree of conservation of kinetic and potential energy of the center of mass translates to a mechanically efficient gait [61]. Some mechanical energy is, however, lost during each step, most notably in the transition between the stance and swing phases. Positive mechanical power, primarily during pre-swing, is required to preserve walking speed during the step-to-step transitions, is the primary determinant of metabolic energy cost, and increases with walking speed [62]. In accordance with this finding, we observed that the push-off action of the plantarflexors during pre-swing accounted for the highest metabolic rates among muscle groups and gait phases, and increased with walking speed. Likewise, the relative cost of the swing phase decreased with walking speed, and is related to muscles’ active contribution to advance the leg. The initiation of swing is produced by the combined action of ankle push-off and hip flexion pull-off actions during pre-swing [63]. At slow walking speeds, less mechanical energy is stored by the plantarflexors in terminal stance and delivered during the push-off action, compared to fast speeds [64], and relatively more hip flexor muscle activation is required for a pull-off action [65]. We found that while the hip flexor muscle activations and associated energy cost during swing did increase with walking speed, the energy costs during stance increased more.

In summary, evaluating the mechanics and metabolic rates of muscles supported by various degrees of experimental observation provides insights into the relationship between muscle-tendon function and metabolic demands and suggests venues to improve our understanding of metabolic rates. By incorporating muscle-tendon parameter individualization and selecting a subset of metabolic energy models from the literature, we could accurately estimate whole-body average metabolic rates and also provide time-series estimates at the muscle group level. Furthermore, our observations lead to the following suggestions:

- Regarding modelling muscle-tendon dynamics, we recommend validating computed passive structures—specifically highly compliant tendons and passive force-length relationship, to describe better time-series trends of muscle activations, fiber lengths, and, thus, metabolic rates.

- Regarding modelling metabolic energy cost, models that account for heat rates based on fiber-type composition and null or small eccentric contraction cost, such as in the BH04 (as modelled in this study) and UM10 models, better describe the increment of the whole-body metabolic rates across walking speeds compared to measured data.

- Among muscle function groups, the ankle plantarflexors account for the highest metabolic cost across walking speeds. Also, plantarflexor activity becomes substantially more costly when walking speed increases, whereas knee and hip extensor, the other muscle groups that account for the highest metabolic rates, are proportionally unaffected by walking speed. This estimation supports the argument that ankle plantarflexion motion is the main driver of metabolic energy cost across walking speeds.

- For metabolic efficiency, it is relatively more important to assist muscle function groups that support the swing phase, specifically hip flexors and adductors, at low rather than fast walking speeds. Assistive devices that support hip flexors have been reported to significantly reduce the metabolic rates near the preferred walking speed [66]. Likely, devices that support hip flexors have more potential to reduce metabolic costs at slow rather than high walking speeds.

- Hip abductors and adductors contribute to approximately one-fifth of the metabolic cost at preferred walking speed; thus, devices that support and assist frontal plane hip muscles seem recommended for walking efficiency. Moreover, supporting these muscles seems more important at low rather than high walking speeds.

Future work in quantifying metabolic rates during motion might be dedicated to increasing the complexity of the musculoskeletal geometry and examining other neuromuscular command models. We assumed that muscle moment arms and muscle-tendon lengths are scaled versions of the generic musculoskeletal model, yet special attention might be warranted for certain muscles whose attachments can be expected to induce large changes in computed muscle dynamics [67]. Modelling assumptions of the muscle-tendon lengths significantly influences the computation of fiber excursion [68] and, thus, muscle work rate, which is a critical value in estimating metabolic rates. Also, we assumed that a single performance criterion solves muscle controls: the sum of squared muscle activations, while it might instead be a product of various criteria simultaneously [69] or experimentally inferred by a more complex muscle control paradigm, such as muscle synergies [70]. Future studies account for high accuracy in the estimated muscle-tendon geometry and muscle control, which might increase confidence in muscle mechanics and metabolic cost predictions.

## Supporting information

S1 Appendix This appendix describes in detail the calibration of passive forces (section A), simulation workflows (section B), comparison of metabolic estimations among simulation workflows (section C), and simulation verification (section D). (PDF)

S1 Fig Passive moment computed with calibrated and generic passive forces across joint ranges of motion. Experimental data was reported by Silder et al. [36]. Positive moments refer to ankle dorsiflexion, knee flexion, and hip flexion, respectively.
(TIFF)

S2 FigMetabolic rates and energy efficiencies in soleus.Metabolic rate ($\dot{E}$) (above) and energy efficiency (below), defined as the ratio between the contractile element work rate and metabolic rate, for a general case of the soleus at several activation levels across normalized fiber lengths (${\tilde{l}}_{M}$), normalized fiber velocities (${\tilde{v}}_{M}$) using six metabolic energy models: Umberger et al. [14] (UM03), Bhargava et al. [12] (BH04), Houdijk et al. [15] (HO06), Lichtwark and Wilson [16] (LW07), Umberger [17] (UM10), and Uchida et al. [18] (UC16). Metabolic rate and energy efficiency for activation levels 0.01, 0.33, 0.66, and 1.00 are shown for ${\tilde{l}}_{M}$ = 1, (plane sections in red color gradient), and for ${\tilde{v}}_{M}$ = 0.1 (plane sections in blue color gradient). Ratio of slow twitch muscle fiber, muscle mass, optimal fiber length, and maximum voluntary contraction are assumed as 0.8, 0.48 kg, 4.5 cm, and 10 [optimal fiber lengths/second], respectively.(TIFF)

S3 Fig Computed and experimental muscle excitations in the simulation workflows. Muscle excitations of all muscle-tendon actuators in the musculoskeletal model at preferred walking speed with four simulation workflows: Minimal muscle effort with generic passive force (GEN), with calibrated passive force (PAS), with calibrated passive force and personalized tendon stiffness (TEN), and EMG-informed with calibrated passive force and personalized tendon stiffness (EMG). Muscle names (plot titles) refer to their abbreviations in the musculoskeletal model: adductor brevis (addbrev), adductor longus (addlong), adductor magnus (addmagDist, addmagIsch, addmagMid, and addmagProx), biceps femoris long head (bflh), biceps femoris short head (bfsh), extensor digitorum longus (edl), extensor hallucis longus (ehl), flexor digitorum longus (fdl), flexor hallucis longus (fhl), gastrocnemius lateralis (gaslat), gastrocnemius medialis (gasmed), gluteus maximus (glmax1, glmax2, and glmax3), gluteus medialis (glmed1, glmed2, and glmed3), gluteus minimus (glmin1, glmin2, and glmin3), gracilis (grac), iliacus, peroneus brevis (perbrev), peroneus longus (perlong), piri, psoas, rectus femoris (recfem), sartorius (sart), semimembranosus (semimem), semitendinosus (semiten), soleus, tensor fasciae latae (tfl), tibialis anterior (tibant), tibialis posterior (tibpost), vastus intermedius (vasint), vastus lateralis (vaslat), and vastus medialis (vasmed). Measured EMG signals were obtained by digitalizing data reported by Perry [41].
(TIFF)

S4 Fig Relative metabolic cost of muscle function groups across speeds in the simulation workflows. Percentage of the energy cost of the ankle dorsiflexors, ankle plantarflexors, knee flexors, knee extensors, hip flexors, hip extensors, hip adductors, hip abductors, hip internal rotators, and hip external rotators relative to the total energy cost in a gait cycle [%] vs. walking speeds with the simulation workflow based on minimal muscle effort with calibrated passive force and personalized tendon stiffness (TEN), using six metabolic energy models: Umberger et al. [14] (UM03), Bhargava et al. [12] (BH04), Houdijk et al. [15] (HO04), Lichtwark and Wilson [16] (LW07), Umberger [17] (UM10), and Uchida et al. [18] (UC16). Individual subjects are illustrated in different colors, and the slope and correlation coefficient from repeated measures correlation is indicated.
(TIFF)

S1 TableComponents of the metabolic energy models.Principal characteristics of work rate and heat rates in six metabolic energy models: Umberger et al. [1] (UM03), Bhargava et al. [2] (BH04), Houdijk et al. [3] (HO06), Lichtwark and Wilson [4] (LW07), Umberger [5] (UM10), and Uchida et al. [6] (UC16). The UM03, UM10, and UC16 models had one expression that described both the activation $\left({\dot{H}}_{A}\right)$ and maintenance $\left({\dot{H}}_{M}\right)$ heat rates.(PDF)

S2 Table Relative metabolic cost of muscle function groups at preferred walking speed. Percentage of the metabolic cost of the ankle dorsiflexors (AD), ankle plantarflexors (AP), knee flexors (KF), knee extensors (KE), hip flexors (HF), hip extensors (HE), hip adductors (HD), hip abductors (HB), hip internal rotators (HI), and hip external rotators (HO) relative to the total energy cost in a gait cycle [%] at preferred walking speed with four simulation workflows: Minimal muscle effort with generic passive force (GEN), with calibrated passive force (PAS), with calibrated passive force and personalized tendon stiffness (TEN), and EMG-informed with calibrated passive force and personalized tendon stiffness (EMG), using six metabolic energy models: Umberger et al. [1] (UM03), Bhargava et al. [2] (BH04), Houdijk et al. [3] (HO04), Lichtwark and Wilson [4] (LW07), Umberger [5] (UM10), and Uchida et al. [6] (UC16).
(PDF)
