# Supplementary material for: Insights into muscle metabolic energetics: Modelling muscle-tendon mechanics and metabolic rates during walking across speeds
Source: PLoS Comput Biol. 2024 Sep 13;20(9):e1012411. doi: 10.1371/journal.pcbi.1012411 (PMC11424009; doi:10.1371/journal.pcbi.1012411)
Supplement: S1 Appendix — (PDF) [file pcbi.1012411.s001.pdf]

Supplementary Material

A) Calibration of passive forces

The calibration of force-length characteristics in the muscle-tendon actuators was implemented to improve the representation of passive moment in the musculoskeletal model compared to experimental observations reported by Silder et al.[1]. The passive force-length relationship was modeled based on OpenSim's Thelen2003Muscle, see Equation A1

$$f_{pas}(\tilde{l}_M) = \frac{e^{\frac{k_{PE}*(\tilde{l}_M - s_0)}{s_M}} - 1}{e^{k_{PE}} - 1} \qquad \text{(Eq. A1)}$$

Where $\tilde{l}_M$ is the normalized fiber length, k_{PE} is the exponential shape factor for the passive force-length relationship, s_0 is the normalized fiber length at which the passive force starts to increase, and s_M is the normalized fiber length, measured from the optimal fiber length ($\tilde{l}_M = 1$), at which maximum force is reached. The generic values for k_{PE}, s_0, and s_M are 4, 1, and 0.6, respectively. We formulated an optimization routine where we selected k_{PE} and s_0 as the optimization variables, the inverse kinematics and inverse dynamics solutions were prescribed, and muscle-tendon actuators of the musculoskeletal model generated a moment that reproduced the joint moments of the inverse dynamic solution. To guarantee the feasibility of the solution, we added ideal non-physiological actuators at each joint, called reserve actuators. These actuators accounted for the joint moments that the muscle-tendon actuators could not reproduce. The objective function to be minimized was defined as the sum of reserve actuators, the equilibrium between the tendon force and the muscle force, and the moment from the muscle-tendon actuators and the inverse dynamics solution were implemented as optimization constraints, see Equation A2-A7

$$J = \int_{t_i}^{t_f} \left(\sum_{j=1}^{J} r_j^2\,(t) \right) dt \qquad \text{(Eq. A2)}$$

Subject to

$$0 = F_T - F_M cos\alpha \qquad \text{(Eq. A3)}$$

$$0 = T_{Mj} - T_{IDj} \qquad \text{(Eq. A4)}$$

Where

$$T_{Mj} = \sum_{i=1}^{N} d_{mi} F_{Tm} + r_j T_{max} \qquad \text{(Eq. A5)}$$

$$F_M = F_M^0 f_{pas}(\tilde{l}_M) \qquad \text{(Eq. A6)}$$

$$F_T = F_M^0 f_t(\tilde{l}_T) \qquad \text{(Eq. A7)}$$

As such, t_i is the initial time of the gait cycle, t_f is the final time of the gait cycle, r_j is the reserve actuator of the joint j, J the total number of joints in one leg of the musculoskeletal model. F_T is the tendon force, modelled as the maximum isometric force F_M^0 multiplied by the tendon force-length characteristic f_t (Eq. A7). F_M is the muscle force, modelled as the maximum isometric force F_M^0 multiplied by the passive force-length characteristic (Eq. A6). T_{Mj} is the total moment generated by the muscle-tendon actuators at joint j, and T_{IDj} is the moment at joint j computed from inverse dynamics. In addition, α is the pennation angle, d_m is the moment arm, N the total number of muscles in one leg of the musculoskeletal model, and T_{max} is the maximum moment of the

reserve actuator. The value of T_{max} is 150 Nm, and the tendon force-length characteristic by modelled as described by Friedl et al. [4].

We added additional constraints in the optimization problem to improve the accuracy of our results. We coupled the optimization variables between muscles with multiple attachment points or that share similar function (see Supplementary table A1). The values of k_{PE} were bound between 3 and 5, and s_0, between 0.8 and 1.2. We used the digitalized data of the passive moments provided by Uhlrich et al. [2]

The joint moment from the muscle-tendon actuators with calibrated passive force better estimated the reported moments by Silder et al. [1] compared to generic passive force values (Supplementary figure A1). In addition, less reliance on reserve actuators was required to reproduce the experimental joint moment. The values of the optimized parameters are provided in Supplementary table A1.

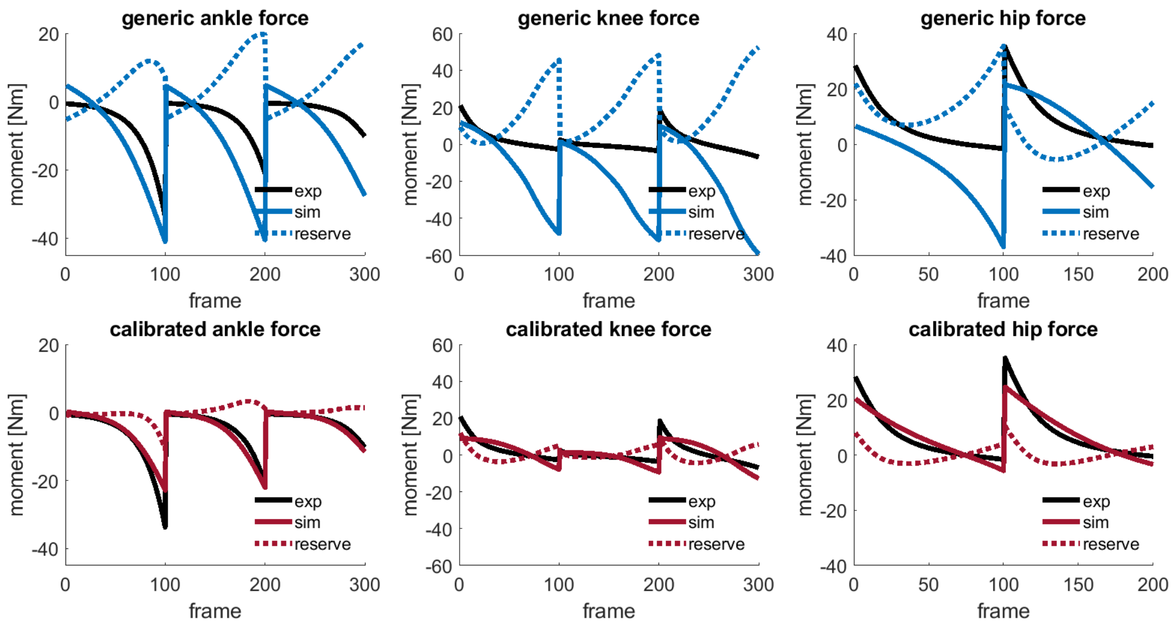

Supplementary figure A1: Passive moment computed with generic and calibrated passive forces at the ankle, knee, and hip joint. Experimental moment was reported by Silder et al. [1]. Reserved actuators were represented by dashed lines. Positive moments refer to ankle dorsiflexion, knee flexion, and hip flexion, respectively.

Supplementary table A1: Values of the calibrated parameters k_{PE} and s_0 from the muscles in the musculoskeletal model.

Muscle	Model name	k_{PE}	s_0
Adductor brevis	addbrev	3.7	0.8
Adductor longus	addlong	3.6	0.8
Adductor magnus	addmagDist	5.0	1.2
	addmagIsch		
	addmagMid		
	addmagProx		
Biceps femoris short head	bfsh	5.0	1.2
Extensor digitorum longus	edl	5.0	1.2
Extensor hallucis longus	ehl		
Flexor digitorum longus	fdl	5.0	1.2
Flexor hallucis longus	fhl		
Gastrocnemius lateral head	gaslat	5.0	1.0
Gastrocnemius medial head	gasmed		
Gluteus maximus	glmax1	5.0	1.2
	glmax2		
	glmax3		
Gluteus medius	glmed1	3.0	0.8
	glmed2		
	glmed3		
Gluteus minimus	glmin1	5.0	1.2
	glmin2		
	glmin3		
Gracilis	grac	5.0	1.2
Iliacus	iliacus	4.0	0.8
Peroneus brevis	perbrev	3.0	1.1
Peroneus longus	perlong		
Piriformis	piri	5.0	1.2
Psoas	psoas	4.9	0.8
Rectus femoris	recfem	5.0	1.2
Sartorius	sart	3.0	0.8
Semimembranosus	semimem	5.0	1.2
Semitendinosus	semiten		
Biceps femoris long head	bflh		
Soleus	soleus	5.0	1.1
Tensor fascia latae	tfl	5.0	1.2
Tibialis anterior	tibant	5.0	1.2
Tibialis posterior	tibpost	5.0	1.2
Vastus intermedius	vasint	5.0	1.2
Vastus lateralis	vaslat		
Vastus medialis	vasmed		

B) Simulation workflows

We performed musculoskeletal simulations with prescribed joint kinematics and dynamics and solved the muscle redundancy as an optimal control problem, where we accounted for various levels of personalization. We started with a scaled generic model and computed muscle recruitment based on minimum effort, then included the calibration of passive forces, personalization of Achilles and quadriceps tendon stiffnesses, and informed the muscle controls with recorded electromyographic signals (EMGs). Calibrated force-length characteristics were obtained from optimizing parameters of the passive force-length curves in a generic musculoskeletal model. Detailed information about the optimization routine is presented in Supplementary material A: Calibration of passive forces. Personalized Achilles and quadriceps tendon stiffness values were computed by optimizing the normalized tendon stiffness values to better match the moment produced by muscle-tendon actuators informed with EMGs and the inverse dynamics solution. In the following subsections, we described the simulations workflows and then the selection of the weights in the objective functions.

1) Muscle redundancy solver based on minimal muscle effort with generic passive forces parameters (GEN), with calibrated passive forces parameters (PAS), and with calibrated passive forces and personalized tendon stiffness (TEN)

The simulation workflows GEN, PAS, and TEN were computed using the same optimal control problem. DeGroote et al. described the computational formulation in detail [4]. In brief, the cost function (J_{EFFORT}) of the dynamic optimization problem consisted in three terms: The first term referred to muscle effort; the second term to the moment produced by reserve actuators; and the third term to the fiber velocity to improve the numerical computation, see Equation B1

$$\boldsymbol{J_{EFFORT} = w_e \int_{t_i}^{t_f} \left(\sum_{i=1}^{N} \frac{\left(e_i^2(t) + a_i^2(t)\right)}{2} \right) dt + w_r \int_{t_i}^{t_f} \left(\sum_{j=1}^{J} r_j^2\,(t) \right) dt + w_v \int_{t_i}^{t_f} \left(\sum_{i=1}^{N} v_i^2(t) \right) dt} \qquad \text{(Eq. B1)}$$

Where t_i is the initial time of the gait cycle, t_f is the final time of the gait cycle, e_i is muscle excitation of the muscle i, a_i is muscle activation of the muscle i, N the total number of muscles in one leg of the musculoskeletal model, r_j is the reserve actuator of the joint j, J the total number of joints in one leg of the musculoskeletal model, v_i is the muscle velocity of the muscle i, and w_e, w_r, and w_v are the weight of the terms related to muscle effort, reserve actuators, and fiber velocities, respectively.

Activation dynamics, contraction dynamics, equilibrium between muscle force and tendon force, and between moment produced by the muscle-tendon actuators and the prescribed joint moments were added as equality constraints in the optimal control problem [4]. The equations that describe the force-length active curve f_{act}, the force-velocity curve, and the force-length passive curve f_{pas}, were the same as described by DeGroote et al. [4].

Parameters of the force-length passive curves, k_{PE} and s_0, and tendon stiffness k_T varied among simulation workflows. The parameter k_{PE} is the exponential shape factor for the passive force-length relationship, s_0 is the normalized fiber length at which the passive force starts to increase, and k_T is the normalized tendon stiffness. In the simulation workflow GEN, k_{PE}, s_0 and k_T were 4, 1, and 35, respectively, for all the muscles. Such value of k_T is equivalent to approximately a tendon strain of 4% at the maximum isometric force. In the simulation workflow PAS, k_{PE} and s_0 had the values computed in the calibration of passive moments from the generic musculoskeletal model, and k_T were 35, for all the muscles. In the simulation workflow TEN, k_{PE} and s_0 had the values computed in the calibration of passive moments from the generic musculoskeletal model, and k_T had the values the personalized tendon stiffness values obtained from the muscle redundancy solver with muscle constrained by EMGs and estimation of tendon stiffness (see next section for details).

2) Muscle redundancy solver based on minimal muscle effort with calibrated passive forces parameters, personalized tendon stiffness, and informed with recorded EMGs (EMG)

The formulation of the simulation workflow EMG was similar to the previous optimal control problem but included constraints in the muscle excitations to inform them of recorded EMGs. Delabastita et al. described the computational formulation in detail [5]. In brief, the cost function (J_{EMG}) of the dynamic optimization problem consisted in four terms: The first term referred to muscle effort; the second term referred to the tracking of the time-series EMG information; the third term to the moment produced by reserve actuators; and the fourth term to the fiber velocity to improve the numerical computation, see Equation B2

$$\boldsymbol{J_{EMG}} = \boldsymbol{w_e} \int_{t_i}^{t_f} \left(\sum_{i=1}^{N} \frac{\left(e_i^2(t)+a_i^2(t)\right)}{2} \right) \boldsymbol{dt} + \boldsymbol{w_t} \int_{t_i}^{t_f} \left(\sum_{k=1}^{K} \left(\boldsymbol{e_k(t)} - \boldsymbol{\hat{e}_{Ek}(t)}\right)^2 \right) \boldsymbol{dt}$$

$$+\boldsymbol{w_r} \int_{t_i}^{t_f} \left(\sum_{j=1}^{J} \boldsymbol{r_j^2}\ \boldsymbol{(t)} \right) \boldsymbol{dt} + \boldsymbol{w_v} \int_{t_i}^{t_f} \left(\sum_{i=1}^{N} \boldsymbol{v_i^2(t)} \right) \boldsymbol{dt} \qquad \text{(Eq. B2)}$$

Subject to

$$-\boldsymbol{e_{lim}} \leq \boldsymbol{e_k(t)} - \boldsymbol{\hat{e}_{Ek}(t)} \leq \boldsymbol{e_{lim}} \qquad \text{(Eq. B3)}$$

Where $\hat{e}_{Ek}$ is the scaled EMGs of the muscle k, K is the total number of EMGs, and w_t is the weight of the term related to EMG tracking. Also, e_{lim} is the EMG tracking bound, which refers to the maximum difference allowed between the estimated excitations $e_k(t)$ and the scaled EMGs $\hat{e}_E(t)$.

Nine muscles were informed with EMGs: biceps femoris long head (BF), semitendinosus (ST), vastus lateralis (VL), medialis (VM), and intermedius (VI), tibialis anterior (TA), gastrocnemius lateralis (GL), and medialis (GM) and soleus (SO). EMG signals were processed and shifted by 40 ms forward in time to account for electromechanical delays [6]. Processed EMGs were scaled to the highest value among all walking trials and a maximum vertical jump trial since we did not perform maximum voluntary contraction trials. Such a procedure led to computing high moment from BF, ST, and TA. As such, we opted to further refine the scaling of the processed EMGs by adding optimization variables in the optimal control problem. A scaling factor was assigned to each muscle actuator informed with EMG, see Equation B3

$$S_{Ek}.\, e_{Ek}(t) = \hat{e}_{Ek}(t) \qquad \text{(Eq. B3)}$$

Where S_{Ek} is the EMG scaling factor of the muscle k, and e_{Ek} is the processed and delayed time-series EMG of the muscle k. The values of the EMG scaling factors were bounded between 0.05 and 2.5. The EMG scaling factor of the GL and GM and of the VI, VL, and VM were coupled as they supported the same muscle function group.

We personalized tendon stiffness in the plantarflexors: GL, GM, and SO and knee extensors: VL, VM, and VI, similarly to the EMG scaling factors. The nonlinear tendon-force relationship was modeled as described by DeGroote et al. [4]. Only the stiffness of the muscles related to the major tendons of the lower limbs: Achilles and quadriceps tendons, were personalized as their tendons are known to be highly compliant. The normalized tendon stiffnesses were optimized by adding optimization variables that scaled a nominal tendon stiffness value, see Equation B4

$$S_{Tl}.\, k_n = k_{Tl} \qquad \text{(Eq. B4)}$$

Where S_{Tl} is the scaling factor of the normalized tendon stiffness of the muscle l, k_{Tl} is the personalized value of the normalized tendon stiffness of the muscle l, and k_n is the nominal value of the normalized tendon stiffness. The nominal tendon stiffness value was 35. The values of the normalized tendon stiffness scaling values S_{Tl} were bounded between 0.1 and 1.2.

The summed of the tendon stiffnesses of the VL, VM, VI, and rectus femoris was regarded as the tendon stiffness of the quadriceps tendon, and the summed of the tendon stiffnesses of the GL, GM, and SO was regarded as the tendon stiffness of the Achilles tendon. In addition, the normalized tendon stiffnesses S_{Tl} of SO, GL, and GM, and of the VI, VL, and VM were coupled. The tendon stiffnesses were coupled under the assumption that muscles

that share insertion attachments, i.e., tendon and aponeuroses, should also present similar mechanical properties in such attachments.

The objective function (Eq. B2) was solved for all 21 walking trials (3 gait cycles at each of 7 walking speeds) simultaneously. Thus, in summary, nine EMG scaling factors and two normalized tendon stiffnesses were optimized per subject while solving for the muscle redundancy.

3) Weights and EMG deviation bounds in the objective functions

In the simulation workflows GEN, PAS, and TEN, we selected the same weights of the terms in their objective functions. The values of w_e, w_r, and w_v were 1, 1000, and 0.01, respectively, as they provided reasonable estimations of muscle excitations and fiber lengths and low reliance on reserve actuators across walking speeds [7]. The use of the reserve actuators was highly discouraged in the objective function due to the large value of their weights, and the influence of the fiber velocity was relatively small.

In the simulation workflow EMG, we performed multiple simulations to select the weights of the terms and the EMG tracking bound in the objective function. Particularly, we analyzed the effect of the weights on the muscle effort w_e, and EMG tracking w_t, and the EMG tracking bound e_{lim} in the computation of reserve actuators, EMG tracking deviation (difference between the estimated excitations $e(t)$ and the scaled EMGs $\hat{e}_E(t)$), tendon stiffness estimation, and fiber lengths. We analyzed two cases for the EMG bounds: tracking the EMGs tightly (e_{lim}=0.01) or loosely (e_{lim}=0.05), and six cases for the combination of the weights for the muscle effort and EMG tracking terms (Supplementary table B1).

The simulation workflow EMG converged in all conditions analyzed. Higher EMG tracking weight w_t than the muscle effort weight w_e reduced the need for reserve actuators and also the EMG tracking deviation (see #T1 vs. #T2 and #L1 vs. #L2 in Supplementary table B1). Moderate EMG tracking weight: w_t=10, led to the low values of reserves actuators (see #T3 and #T4, and #L3 and #L4 vs. others in Supplementary table B1). High EMG tracking weights: w_t=100 and w_t=1000, did not lead to a further decrement in the EMG tracking deviation compared to moderate EMG tracking weight (see #T3 and #T4 vs. #T5 and #T6, and #L3 and #L4 vs. #L5 and #L6 in Supplementary table B1). Consequently, we found that simulations with moderate EMG tracking weight w_t=10 (#T3 and #T4) led to the best outcomes for reserve actuators and EMG tracking deviation.

The values of the estimated Achilles tendon stiffness with thigh EMG tracking bounds (e_{lim}=0.01) were within the recorded values measured in vivo by Stenroth et al. [8], which vary between 141 N/mm to 170 N/mm (see #T1 to #T6 in Supplementary table B1). In addition, it led to a good agreement between estimated and digitalized fiber lengths across walking speeds were obtained (Supplementary figure B1). The values of the estimated quadriceps tendon stiffness were not possible to compare experimentally as, to the best of our knowledge, no study has reported its value in vivo, and the musculoskeletal did not model the patellar and quadriceps tendon independently. Nonetheless, we indirectly validated it by observing which tendon stiffness allowed us to model fiber lengths of VL better. In this regard, simulation with thigh EMG tracking bounds (e_{lim}=0.01) and moderate EMG tracking weight (see #T3 and #T4 in Supplementary table B1) estimated a nearly isometric contraction pattern during loading response at preferable walking speed (Supplementary figure B1), which has been reported by Bohm et al. [9] and Chleboun et al. [10]. In this regard, we opted for a simulation that allows the muscle-tendon actuators to closely follow the EMGs with low reliance on reserve actuators and estimate Achilles tendon and fiber lengths compared to experimental findings available in the literature: EMG tracking weight w_t, muscle effort weight w_e, and EMG tracking bound e_{lim} were 10.0, 0.10, and 0.01, respectively (#T4 in Supplementary table B1).

We did not choose EMG with loose EMG tracking bound (e_{lim}=0.05) as the computation of fiber lengths and muscle excitations were quite sensitive to the simulation setups. The simulation EMG with thigh EMG tracking bound (e_{lim}=0.01) was less sensitive and yielded a more realistic estimation of fiber lengths. In addition, simulations #T3 and #T4 derived similar muscle mechanics and yielded the same conclusions in this study.

Supplementary table B1: Simulation setups and outcomes using different EMG tracking weights, muscle effort weights, and EMG tracking bounds of the EMG-informed simulation

**Simulation setup**					**Simulation outcomes**									
**Type**		**Bound [ ]**	**Weight [ ]**		**Solution**	**Reserve actuators [Nm]***			**EMG Tracking deviation [ ]****				**Tendon stiffness [N/mm]*****	
**Scheme**	**#**	EMG deviation e_{lim}	EMG tracking w_t	Muscle effort w_e		Ankle joint	Knee joint	Hip joint	Knee flexors	Knee extensors	Dorsiflexors	Plantarflexors	Achilles	Quadriceps
EMG	T1	±0.01 (Thigh)	0.10	1.00	Yes	3.73	0.97	0.07	0.01	0.01	0.01	0.01	169.40	132.10
	T2		1.00	0.10	Yes	3.17	0.69	0.02	0.00	0.01	0.01	0.01	158.60	110.00
	T3		10.00	0.01	Yes	3.07	0.67	0.01	0.00	0.00	0.00	0.00	151.40	105.70
	**T4**		**10.00**	**0.10**	**Yes**	**3.16**	**0.66**	**0.02**	**0.00**	**0.00**	**0.00**	**0.01**	**158.80**	**109.20**
	T5		100.00	0.10	Yes	3.18	0.70	0.02	0.00	0.00	0.00	0.00	159.80	109.60
	T6		1000.00	0.10	Yes	3.28	0.62	0.05	0.00	0.00	0.00	0.00	144.20	125.90
	L1	±0.05 (Loose)	0.10	1.00	Yes	0.54	0.12	0.05	0.02	0.03	0.03	0.04	159.90	192.90
	L2		1.00	0.10	Yes	0.32	0.04	0.01	0.00	0.02	0.02	0.02	129.80	109.50
	L3		10.00	0.01	Yes	0.32	0.02	0.00	0.00	0.00	0.00	0.01	108.90	102.00
	L4		10.00	0.10	Yes	0.33	0.04	0.01	0.00	0.01	0.00	0.01	137.90	129.30
	L5		100.00	0.10	Yes	0.48	0.10	0.01	0.00	0.00	0.00	0.00	138.90	123.40
	L6		1000.00	0.10	Yes	1.20	0.37	0.02	0.00	0.00	0.00	0.00	138.30	125.70

* Average of the maximum values of the reserve actuators among all the trials and subjects. The average reserve actuators at the hip in the three planes are presented.

** average of the deviations between the estimated excitations $e(\boldsymbol{t})$ and the scaled EMGs $\hat{\boldsymbol{e}}_{\boldsymbol{E}}(\boldsymbol{t})$ among all the trials and subjects. The average of the EMG tracking deviations at the knee flexors: BF, and ST, knee extensors: VL, VM, and VI, dorsiflexor: TA, and plantarflexors: GL, GM, and SO, are presented

*** average of the un-normalized values of the tendon stiffness among all subjects. Unnormalized values of each muscle were computed as the normalized values multiplied by the maximum isometric force and divided by the tendon slack length. Tendon stiffness of the Achilles tendon is computed as the sum of the tendon stiffness of the GL, GM, and SO, and the tendon stiffness of the quadriceps tendon as the sum of the tendon stiffness of the VL, VM, VI, and rectus femoris.

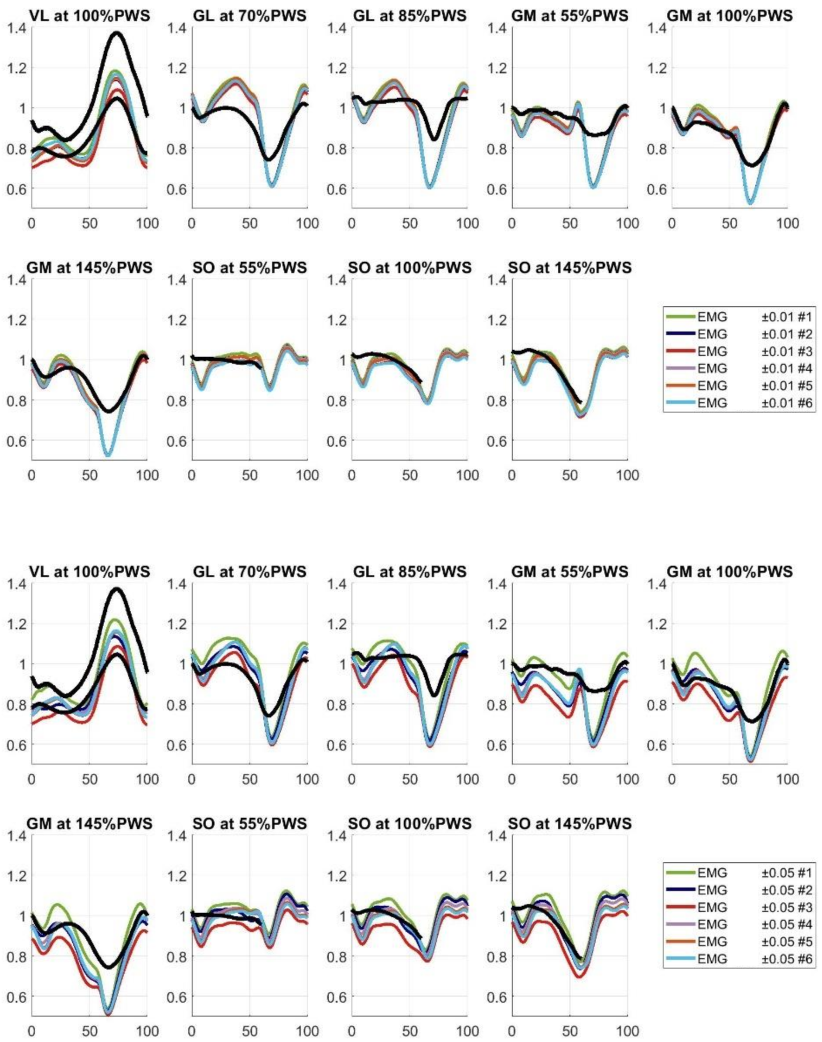

Supplementary figure B1: Normalized fiber lengths vs. gait cycle of vastus lateralis (VL), gastrocnemius lateralis (GL), gastrocnemius medialis (GM), and soleus (SO) at various walking speeds using different simulation setups. Normalized fiber lengths represented the average values among all subjects. Experimental values of fiber lengths were obtained by digitalizing previously reported experimental findings [9–14]. Experimental fiber lengths were normalized based on average values reported from a muscle architecture data set (Ward et al., 2009) if experimental studies did not provide normalized values.

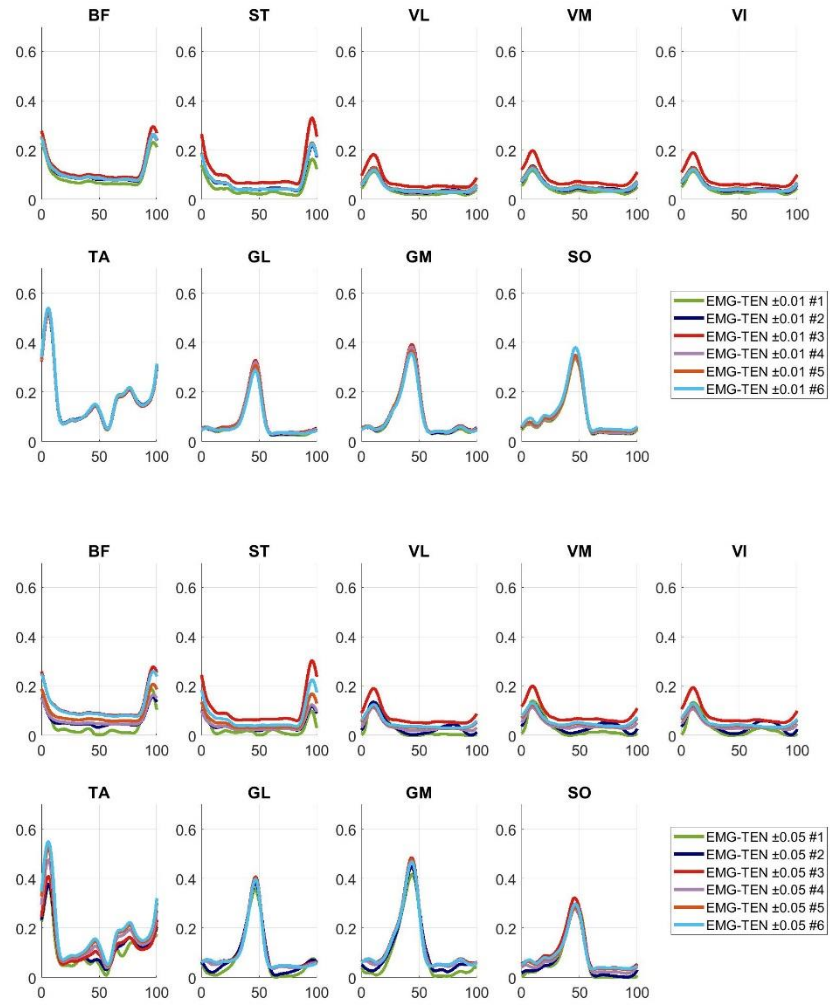

Supplementary figure B2: Muscle excitations vs. gait cycle of biceps femoris long head (BF), semitendinosus (ST), vastus lateralis (VL), vastus medialis (VM), tibialis anterior (TA), gastrocnemius lateralis (GL), gastrocnemius medialis (GM) and soleus (SO) at preferable walking speed using different simulation setups. Muscle excitations represented the average values among all subjects.

C) Comparison of metabolic estimations among simulation workflows

We selected the simulation framework TEN as the one with the highest degree of accuracy. Nonetheless, we also observed some interesting trends among the simulation workflows and the estimation of the metabolic rates. In this section, we summarize the most relevant findings in such comparisons.

The relative cost of the gait phase is greatly modulated by calibrating passive forces

At the preferred walking speed, the simulation workflows GEN and EMG estimated that the swing phase nearly accounted for one-third of the total energy cost during the gait cycle (Supplementary figure C1). The simulation workflows PAS and TEN estimated a lower relative cost of the swing phase, slightly more than one-quarter of the total energy cost. Also, the metabolic cost models almost estimated the same relative cost among such simulation workflows. In the simulation workflow TEN, the relative cost of the swing phase was similar between metabolic cost models.

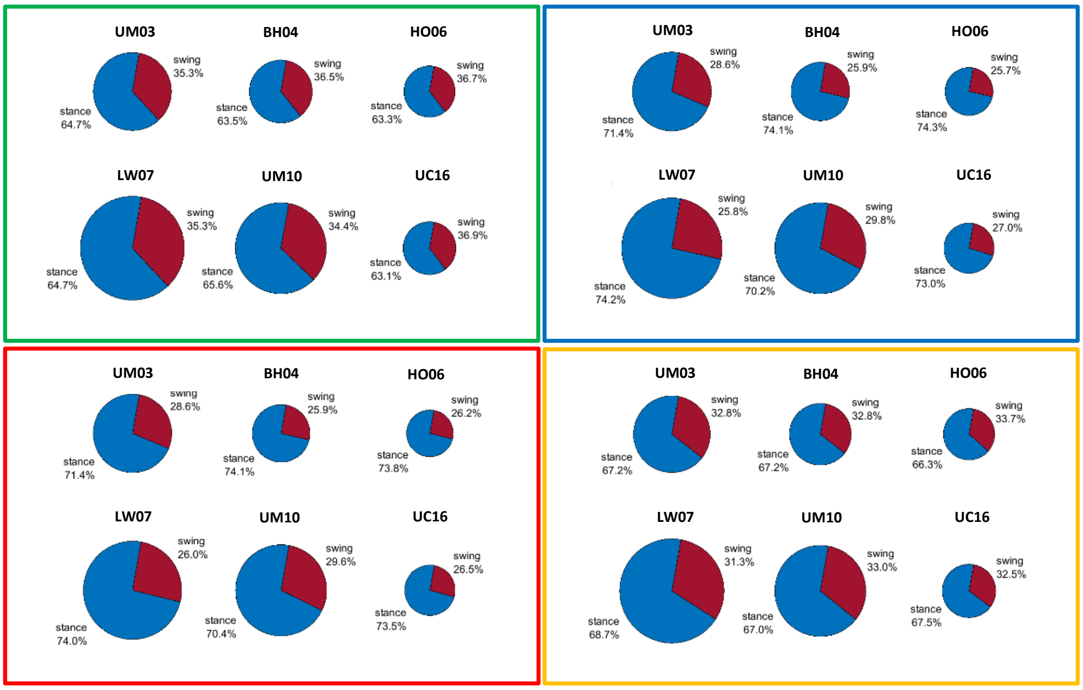

Supplementary figure C1: Cost of the stance and swing phase relative to the total energy cost in a gait cycle during preferred walking speed with four simulation workflows: Minimal muscle effort with generic passive force (GEN), with calibrated passive force (PAS), with calibrated passive force and personalized tendon stiffness (TEN), and EMG-informed with calibrated passive force and personalized tendon stiffness (EMG), using six metabolic cost models: Umberger et al. (UM03), Bhargava et al. (BH04), Houdijk et al. (HO06), Lichtwark and Wilson (LW07), Umberger (UM10) and Uchida et al. (UC16). Pie chart areas are scaled based on the total energy cost in each simulation workflow.

The relative cost of the stance phase increased across walking speeds for all the simulation workflows, yet the magnitude of increment was somewhat decreased with a higher level of personalization. We found a significant ($p<0.05$) yet moderate correlation between the relative cost of gait phases and walking speed in each simulation workflow (Supplementary figure C2). The correlation slope was the highest in the generic simulation workflow (GEN) and the lowest in the EMG-informed simulation workflow (EMG). In the simulation workflow with the best accuracy (TEN), we found that the correlation slope's magnitudes depended on the metabolic cost models.

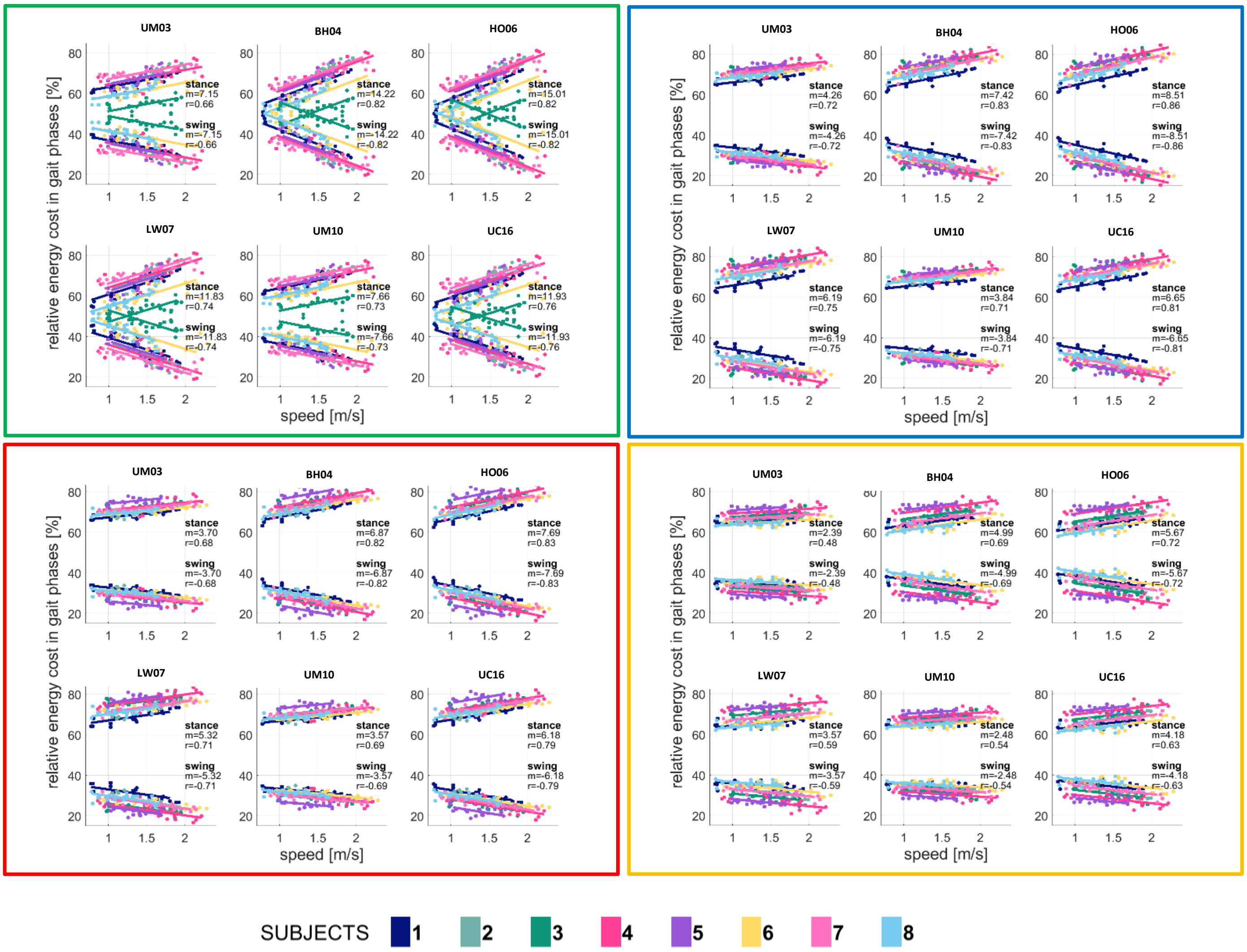

Supplementary figure C2: The cost of the stance and swing phases relative to the total energy cost in a gait cycle (in percentage) vs. walking speeds with four simulation workflows: Minimal muscle effort with generic passive force (GEN), with calibrated passive force (PAS), with calibrated passive force and personalized tendon stiffness (TEN), and EMG-informed with calibrated passive force and personalized tendon stiffness (EMG), using the six metabolic cost models: Umberger et al. (UM03), Bhargava et al. (BH04), Houdijk et al. (HO06), Lichtwark and Wilson (LW07), Umberger (UM10) and Uchida et al. (UC16). Individual subjects are illustrated in different colors, and the slope and correlation coefficient from repeated measures correlation is indicated. The P-value was <0.05 for all correlations

Further insights into the muscle mechanics and energetics across levels of personalization

Calibration of passive forces improved the correlation between estimated and recorded whole-body average metabolic rates compared to the generic simulation workflow. The high correlation in the simulation workflow GEN (0.93-0-96) might be attributed to accurately estimating the salient features of the excitations in most musculoskeletal model muscles. The simulation workflow PAS modeled muscle excitations at the hip and knee joint muscles better, which also resulted in improving the correlation (0.95-0-97). The calibration of passive forces primarily increased the compliance at the hip and knee joints, drastically decreasing the knee flexor activations during the pre-and initial swing and the hip flexors during the entire gait cycle. In addition, it enabled the adductor magnus, bicep femoris long head, gluteus maximus, semimembranosus, and semitendinosus to generate active force during the beginning and end of the gait cycle. Our findings suggest validating estimates of passive forces if muscle excitations and metabolic costs are an outcome of interest.

Calibration of passive force curves might be required to model the muscle force-generation capacity and metabolic cost accurately. Passive forces have been commonly customized by modifying the optimal fiber and/or tendon slack lengths [15–17], as such parameters offset the passive force generation relative to the fiber length. However, those parameters are also related to the force-generation capacity of the muscles and thus might lead to improperly estimated muscle fiber excursions. For instance, Lai et al. [16] modified the optimal fiber lengths and tendon slack lengths of the model proposed by Rajagopal et al. [18] to better describe the passive force generation. This modification led to a better representation of muscle excitations but shorter fiber lengths, which did not agree with experimental observations [7]. In this regard, our results support incorporating the calibration of passive force curves to improve the estimation of muscle activations without affecting the operating range of the muscles. An accurate representation of passive force generation might provide reliable insights into the metabolic cost and neuromuscular strategies of muscle-tendon stiffness modulation [17,19].

Personalization of tendon compliance improved the estimation of fiber lengths compared to the generic simulation workflow and modulated the metabolic rate peaks across walking speeds. Personalized tendons were more compliant and better described the fiber lengths in the gastrocnemius, soleus, and vastus lateralis compared to experimental observations from ultrasound imaging [9–14]. Changes in muscle excursions in the plantarflexors reduced the muscle contraction velocities related to higher force generation capacity and metabolic muscle efficiency [20]. Consequently, it caused a relatively large reduction in the metabolic rate peak of the plantarflexors in all the metabolic cost models in the simulation workflow TEN compared to GEN. We did not observe such a reduction in the vastus muscles in the simulation workflow TEN, likely because such muscles operated at a lower operating range, i.e., at a lower normalized fiber length, even though muscle fibers contracted somewhat isometrically during the loading response. Operating at a lower range decreased the muscle-force generation capacity and thus increased muscle activations and metabolic cost. By assuming an optimal fiber length, we can estimate that the operating range of vastus lateralis during loading response is in ascending limb [10] or near the plateau [9], and such distinction might influence our metabolic cost rate predictions. The metabolic rate peak reduction due to high tendon compliance seems plausible based on simulation workflow EMG, where the knee extensors peak is decreased. To the best of our knowledge, it has not been observed that such nearly isometric contraction during loading response is prevalent across walking speeds or also present in the vastus medialis, which complicated the further validation of our estimates. Nonetheless, the energy-saving mechanism estimated at the plantarflexors in our simulation workflow TEN aligns with previous experimental observations [21] and seems plausible in the knee extensors. Further experimental observations in the vastus muscles might refine our estimates of muscle fibers and metabolic rates.

D) Simulation verification

Here, we provide information about marker error trajectories as well as the computed inverse kinematics and inverse dynamics.

The RMS error between recorded marker trajectories and virtual marker trajectories in the musculoskeletal model had a mean value of 2.1 cm across lower limbs. The maximum marker error was found in the first metatarsal marker and was 8.0 cm. Such a value was observed mainly during the swing phase and is likely to occur since ankle eversion/inversion was locked, as also found in a previous study[22].

Joint angles and moments resemblance values reported in previous studies[23,24]. Joint angles and moments' trajectories are largely preserved across walking speeds, but the peak magnitudes are speed-dependent (Supplementary figure D1). The peak dorsiflexion angle is lower at high speeds than at slow speeds, while peak knee flexion, hip flexion, and hip extension angles increase with increasing speeds.

Relatively low marker trajectory errors and resemblance of joint angle and moment trajectories compared to prior studies provide confidence that the inverse kinematics and dynamics solutions are accurate for our simulation study.

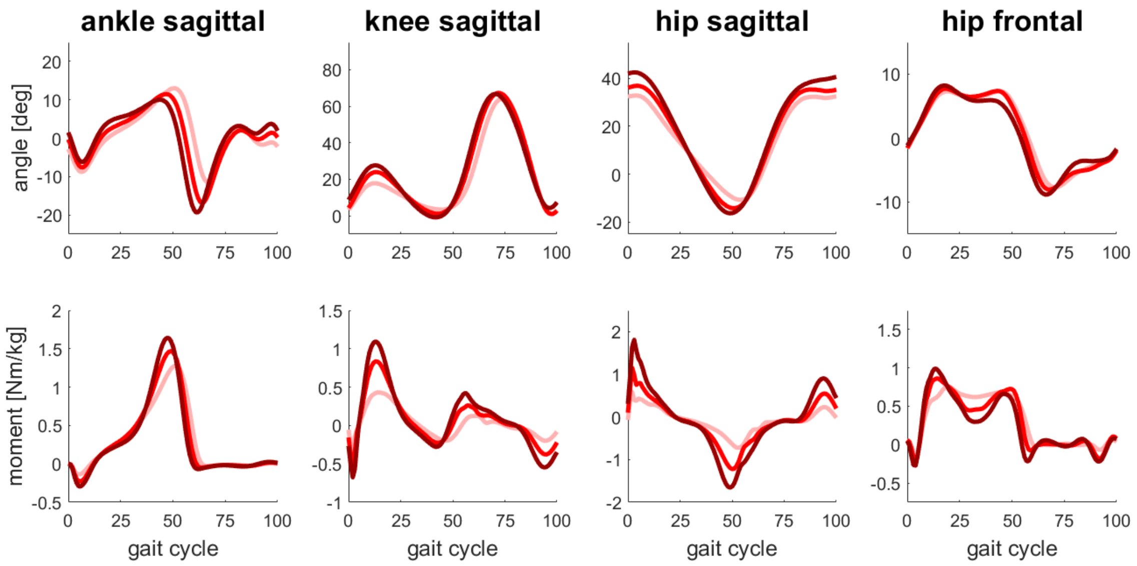

Supplementary figure D1: Joint angles and moments of the lower limbs at 55% PWS (light red), 100% PWS (normal red), and 145% PWS (dark red). Joint angles and moments represented the average values among all subjects, and joint moments were scaled by their mass.

Bibliography

1. Silder, A., Whittington, B., Heiderscheit, B. & Thelen, D. G. Identification of passive elastic joint moment-angle relationships in the lower extremity. *J Biomech* **40**, 2628–2635 (2007).

2. Uhlrich, S. D., Jackson, R. W., Seth, A., Kolesar, J. A. & Delp, S. L. Muscle coordination retraining inspired by musculoskeletal simulations reduces knee contact force. *Sci Rep* **12**, 1–13 (2022).

3. Perry, J. *Gait Analysis: Normal and Pathological Function*. *SLACK Incorporated, New Jersey* (SLACK Incorporated, Thorofare, 1992).

4. De Groote, F., Kinney, A. L., Rao, A. V. & Fregly, B. J. Evaluation of Direct Collocation Optimal Control Problem Formulations for Solving the Muscle Redundancy Problem. *Ann Biomed Eng* **44**, 2922–2936 (2016).

5. Delabastita, T., Afschrift, M., Vanwanseele, B. & De Groote, F. Ultrasound-Based Optimal Parameter Estimation Improves Assessment of Calf Muscle–Tendon Interaction During Walking. *Ann Biomed Eng* **48**, 722–733 (2020).

6. Lloyd, D. G. & Besier, T. F. An EMG-driven musculoskeletal model to estimate muscle forces and knee joint moments in vivo. *Journal of Biomechanics* **36**, 765–776 (2003).

7. Luis, I., Afschrift, M., De Groote, F. & Gutierrez-Farewik, E. M. Evaluation of musculoskeletal models, scaling methods, and performance criteria for estimating muscle excitations and fiber lengths across walking speeds. *Front Bioeng Biotechnol* **10**, 1–16 (2022).

8. Stenroth, L., Peltonen, J., Cronin, N. J., Sipilä, S. & Finni, T. Age-related differences in Achilles tendon properties and triceps surae muscle architecture in vivo. *J Appl Physiol* **113**, 1537–1544 (2012).

9. Bohm, S., Marzilger, R., Mersmann, F., Santuz, A. & Arampatzis, A. Operating length and velocity of human vastus lateralis muscle during walking and running. *Sci Rep* **8**, 1–10 (2018).

10. Chleboun, G. S., Busic, A. B., Graham, K. K. & Stuckey, H. A. Fascicle length change of the human tibialis anterior and vastus lateralis during walking. *Journal of Orthopaedic and Sports Physical Therapy* **37**, 372–379 (2007).

11. Hamard, R. *et al.* Does different activation between the medial and the lateral gastrocnemius during walking translate into different fascicle behavior? *Journal of Experimental Biology* **224**, 1–9 (2021).

12. Farris, D. J. & Raiteri, B. J. Elastic ankle muscle-tendon interactions are adjusted to produce acceleration during walking in humans. *Journal of Experimental Biology* **220**, 4252–4260 (2017).

13. Farris, D. J. & Sawicki, G. S. Human medial gastrocnemius force-velocity behavior shifts with locomotion speed and gait. *Proc Natl Acad Sci U S A* **109**, 977–982 (2012).

14. Lai, A. *et al.* In vivo behavior of the human soleus muscle with increasing walking and running speeds. *J Appl Physiol* **118**, 1266–1275 (2015).

15. Delp, S. L. *et al.* An Interactive Graphics-Based Model of the Lower Extremity to Study Orthopaedic Surgical Procedures. *IEEE Trans Biomed Eng* **37**, 757–767 (1990).

16. Lai, A. K. M., Arnold, A. S., Wakeling, J. M., Biology, E. & Station, C. F. Why are antagonist muscles co-activated in my simulation? A musculoskeletal model for analysing human locomotor tasks. *Ann Biomed Eng* **45**, 2762–2774 (2018).

17. Cop, C. P., Schouten, A. C., Koopman, B. & Sartori, M. Electromyography-driven model-based estimation of ankle torque and stiffness during dynamic joint rotations in perturbed and unperturbed conditions. *Journal of Biomechanics* **145**, 111383 (2022).

18. Rajagopal, A. *et al.* Full-Body Musculoskeletal Model for Muscle-Driven Simulation of Human Gait. *IEEE Trans Biomed Eng* **63**, 2068–2079 (2016).

19. Sartori, M., Maculan, M., Pizzolato, C., Reggiani, M. & Farina, D. Modeling and simulating the neuromuscular mechanisms regulating ankle and knee joint stiffness during human locomotion. *J Neurophysiol* **114**, 2509–2527 (2015).

20. Smith, N. P., Barclay, C. J. & Loiselle, D. S. The efficiency of muscle contraction. *Prog Biophys Mol Biol* **88**, 1–58 (2005).

21. Roberts, T. J. & Azizi, E. Flexible mechanisms: the diverse roles of biological springs in vertebrate movement. *The Journal of experimental biology* **214**, 353–361 (2011).

22. Dembia, C. L., Silder, A., Uchida, T. K., Hicks, J. L. & Delp, S. L. Simulating ideal assistive devices to reduce the metabolic cost of walking with heavy loads. *PLoS One* **12**, (2017).

23. Farris, D. J. & Sawicki, G. S. The mechanics and energetics of human walking and running: A joint level perspective. *J R Soc Interface* **9**, 110–118 (2012).

24. Winter, D. A. *KINEMATIC AND KINETIC PATTERNS IN HUMAN GAIT: VARIABILITY AND COMPENSATING EFFECTS*. *Human Movement Science* vol. 3 (1984).

List of legends of the supporting Information

S1 Fig: Passive moment computed with calibrated and generic passive forces across joint ranges of motion.

S2 Fig: Metabolic rates and energy efficiencies in soleus.

S3 Fig: Computed and experimental muscle excitations in the simulation workflows.

S4 Fig: Relative metabolic cost of muscle function groups across speeds in the simulation workflows.

S1 Table: Components of the metabolic energy models.

S2 Table: Relative metabolic cost of muscle function groups at preferred walking speed.
