# Supplementary figures and images for: Insights into muscle metabolic energetics: Modelling muscle-tendon mechanics and metabolic rates during walking across speeds
Source: PLoS Comput Biol. 2024 Sep 13;20(9):e1012411. doi: 10.1371/journal.pcbi.1012411 (PMC11424009; doi:10.1371/journal.pcbi.1012411)

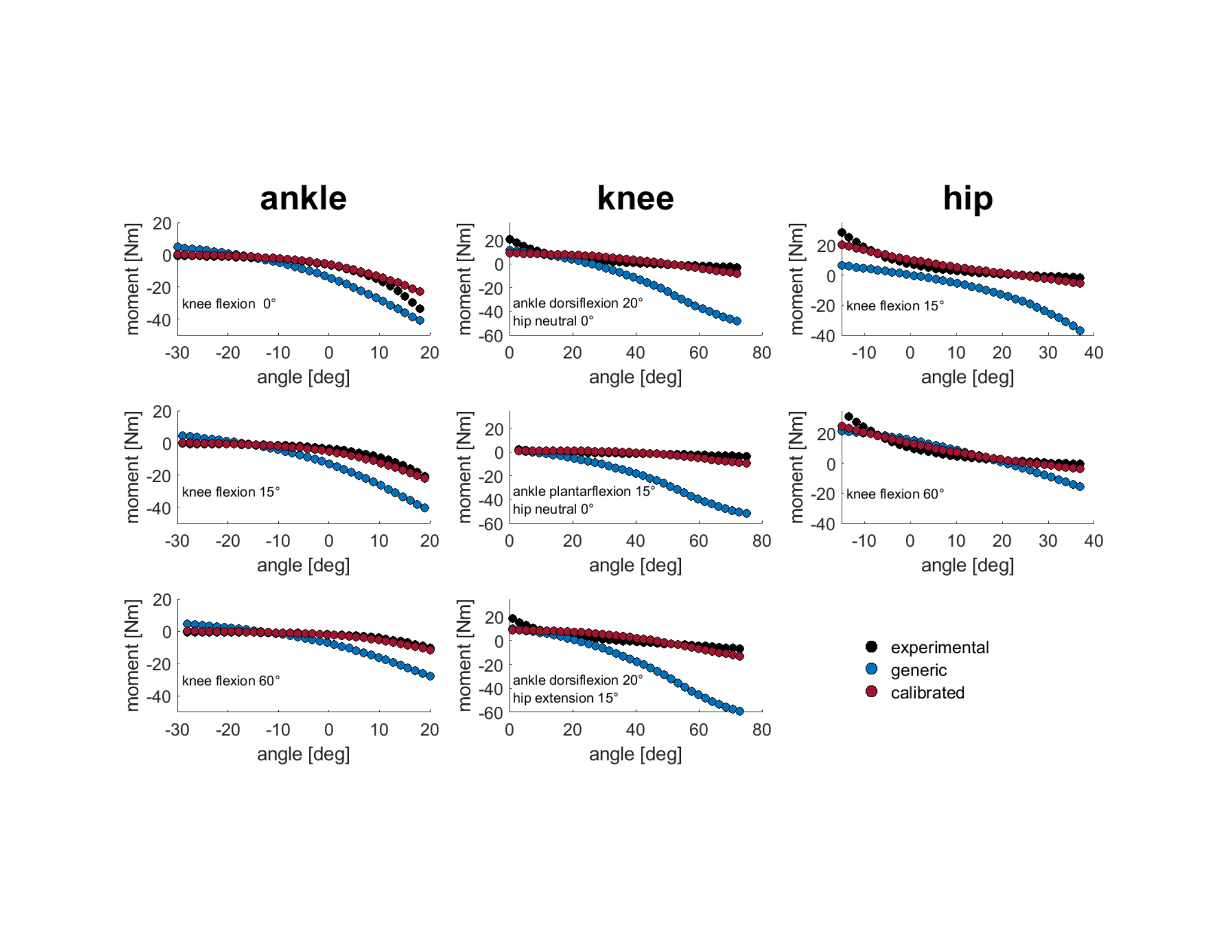

Supplement: S1 Fig — Experimental data was reported by Silder et al. [36]. Positive moments refer to ankle dorsiflexion, knee flexion, and hip flexion, respectively. (TIFF) [file pcbi.1012411.s002.tiff]

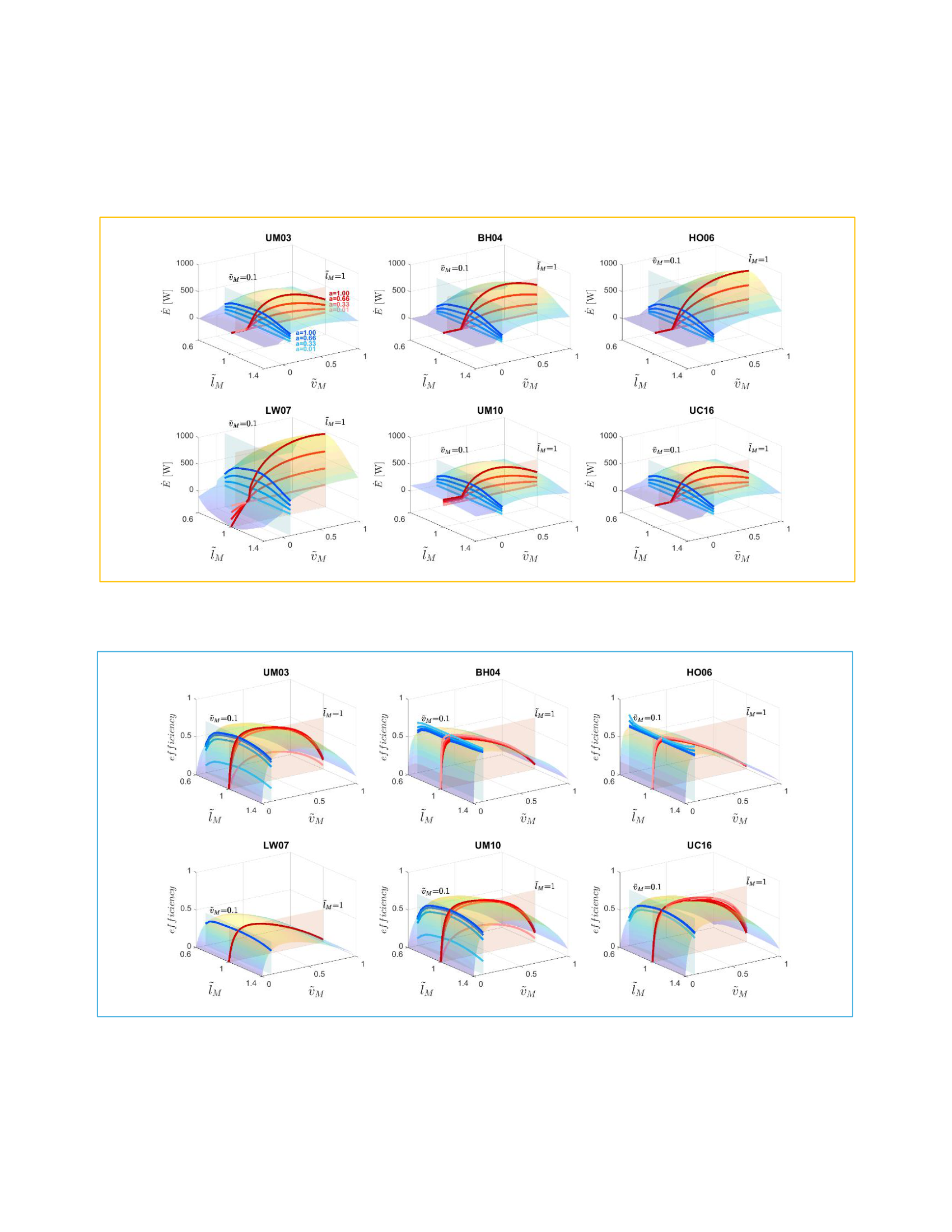

Supplement: S2 Fig — Metabolic rate (E˙) (above) and energy efficiency (below), defined as the ratio between the contractile element work rate and metabolic rate, for a general case of the soleus at several activation levels across normalized fiber lengths (l˜M), normalized fiber velocities (v˜M) using six metabolic energy models: Umberger et al. [14] (UM03), Bhargava et al. [12] (BH04), Houdijk et al. [15] (HO06), Lichtwark and Wilson [16] (LW07), Umberger [17] (UM10), and Uchida et al. [18] (UC16). Metabolic rate and energy efficiency for activation levels 0.01, 0.33, 0.66, and 1.00 are shown for l˜M = 1, (plane sections in red color gradient), and for v˜M = 0.1 (plane sections in blue color gradient). Ratio of slow twitch muscle fiber, muscle mass, optimal fiber length, and maximum voluntary contraction are assumed as 0.8, 0.48 kg, 4.5 cm, and 10 [optimal fiber lengths/second], respectively. (TIFF) [file pcbi.1012411.s003.tiff]

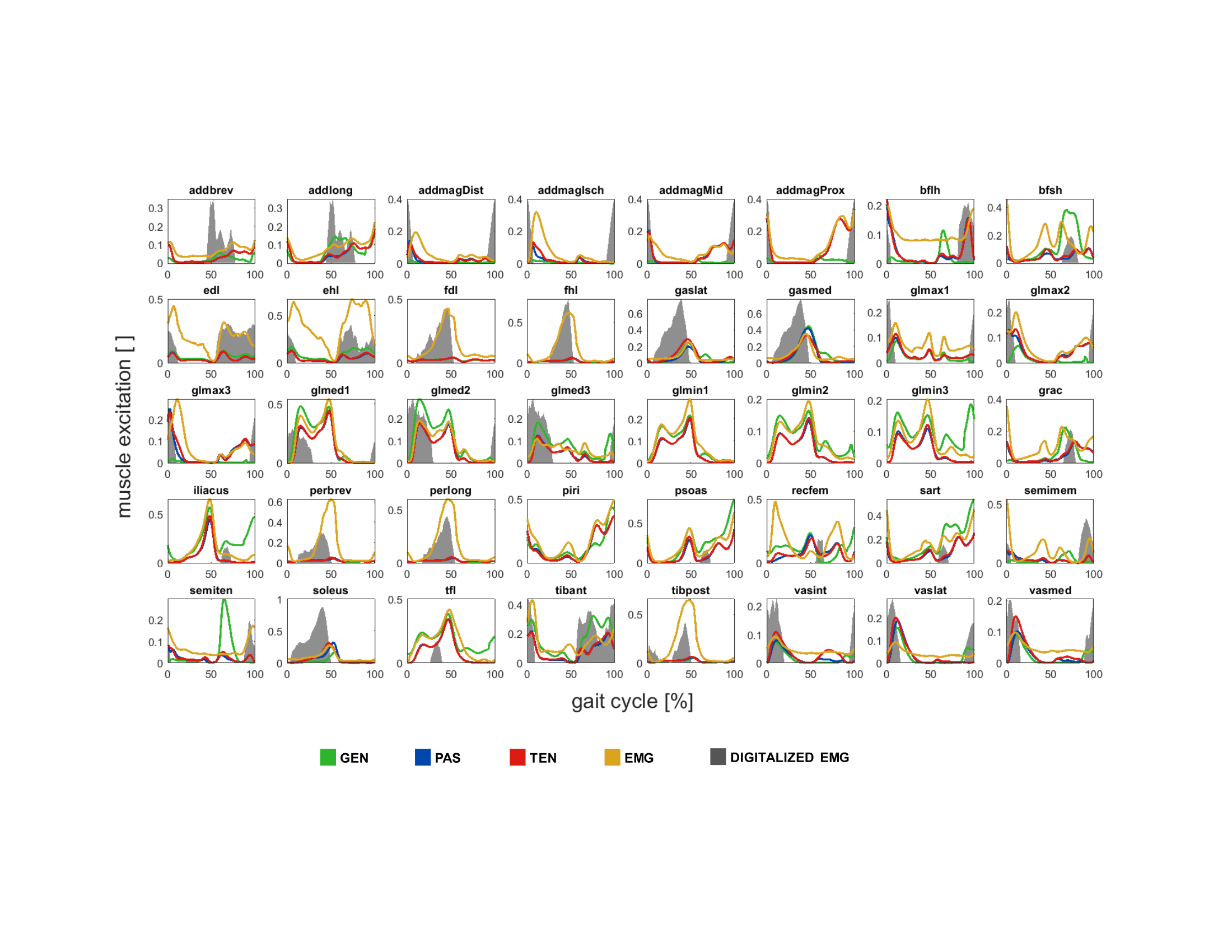

Supplement: S3 Fig — Muscle excitations of all muscle-tendon actuators in the musculoskeletal model at preferred walking speed with four simulation workflows: Minimal muscle effort with generic passive force (GEN), with calibrated passive force (PAS), with calibrated passive force and personalized tendon stiffness (TEN), and EMG-informed with calibrated passive force and personalized tendon stiffness (EMG). Muscle names (plot titles) refer to their abbreviations in the musculoskeletal model: adductor brevis (addbrev), adductor longus (addlong), adductor magnus (addmagDist, addmagIsch, addmagMid, and addmagProx), biceps femoris long head (bflh), biceps femoris short head (bfsh), extensor digitorum longus (edl), extensor hallucis longus (ehl), flexor digitorum longus (fdl), flexor hallucis longus (fhl), gastrocnemius lateralis (gaslat), gastrocnemius medialis (gasmed), gluteus maximus (glmax1, glmax2, and glmax3), gluteus medialis (glmed1, glmed2, and glmed3), gluteus minimus (glmin1, glmin2, and glmin3), gracilis (grac), iliacus, peroneus brevis (perbrev), peroneus longus (perlong), piri, psoas, rectus femoris (recfem), sartorius (sart), semimembranosus (semimem), semitendinosus (semiten), soleus, tensor fasciae latae (tfl), tibialis anterior (tibant), tibialis posterior (tibpost), vastus intermedius (vasint), vastus lateralis (vaslat), and vastus medialis (vasmed). Measured EMG signals were obtained by digitalizing data reported by Perry [41]. (TIFF) [file pcbi.1012411.s004.tiff]

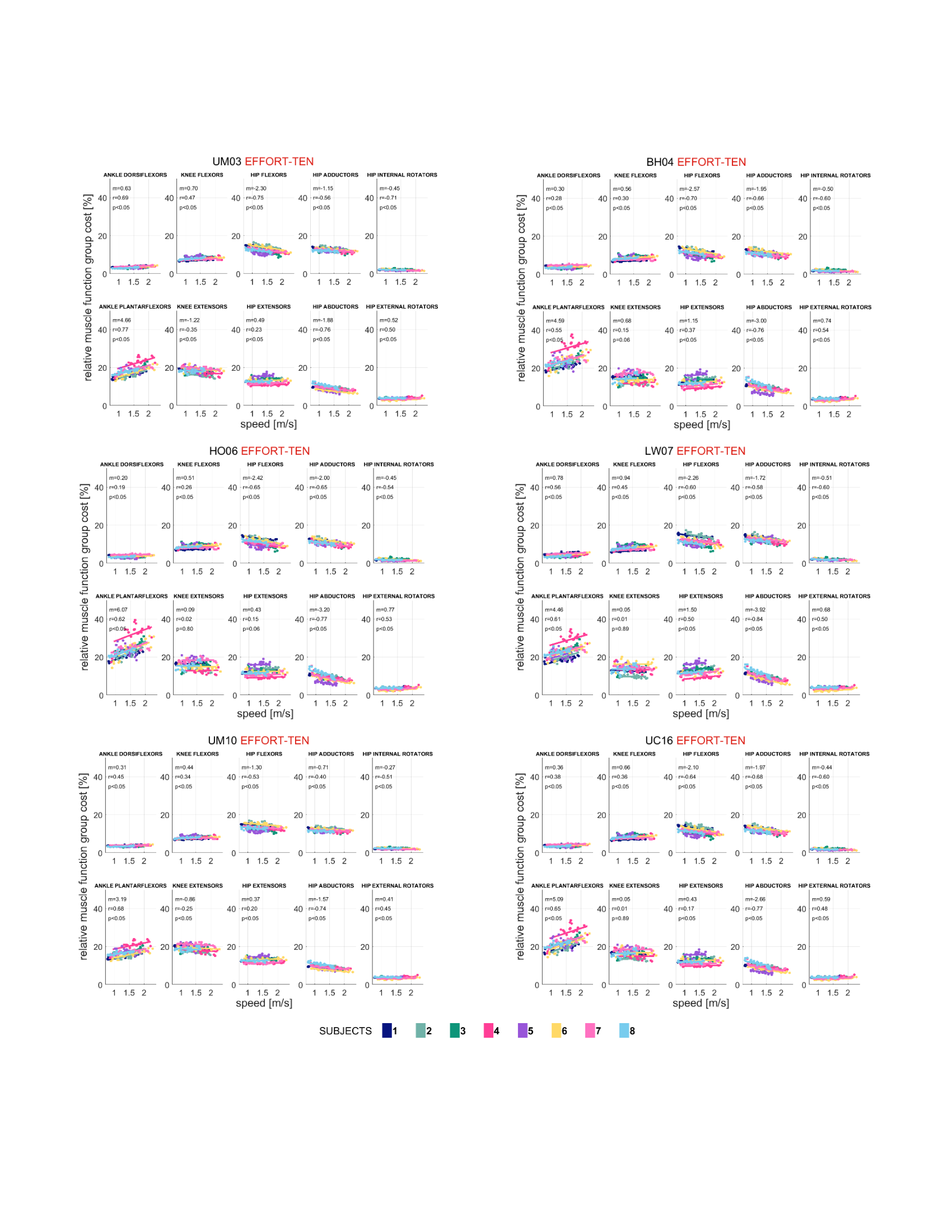

Supplement: S4 Fig — Percentage of the energy cost of the ankle dorsiflexors, ankle plantarflexors, knee flexors, knee extensors, hip flexors, hip extensors, hip adductors, hip abductors, hip internal rotators, and hip external rotators relative to the total energy cost in a gait cycle [%] vs. walking speeds with the simulation workflow based on minimal muscle effort with calibrated passive force and personalized tendon stiffness (TEN), using six metabolic energy models: Umberger et al. [14] (UM03), Bhargava et al. [12] (BH04), Houdijk et al. [15] (HO04), Lichtwark and Wilson [16] (LW07), Umberger [17] (UM10), and Uchida et al. [18] (UC16). Individual subjects are illustrated in different colors, and the slope and correlation coefficient from repeated measures correlation is indicated. (TIFF) [file pcbi.1012411.s005.tiff]
