# Supplementary material for: Insights into muscle metabolic energetics: Modelling muscle-tendon mechanics and metabolic rates during walking across speeds
Source: PLoS Comput Biol. 2024 Sep 13;20(9):e1012411. doi: 10.1371/journal.pcbi.1012411 (PMC11424009; doi:10.1371/journal.pcbi.1012411)
Supplement: S1 Table — Principal characteristics of work rate and heat rates in six metabolic energy models: Umberger et al. [1] (UM03), Bhargava et al. [2] (BH04), Houdijk et al. [3] (HO06), Lichtwark and Wilson [4] (LW07), Umberger [5] (UM10), and Uchida et al. [6] (UC16). The UM03, UM10, and UC16 models had one expression that described both the activation (H˙A) and maintenance (H˙M) heat rates. (PDF) [file pcbi.1012411.s006.pdf]

S1 Table: Components of the metabolic energy models. Principal characteristics of work rate and heat rates in six metabolic energy models: Umberger et al. [1] (UM03), Bhargava et al. [2] (BH04), Houdijk et al. [3] (HO06), Lichtwark and Wilson [4] (LW07), Umberger [5] (UM10), and Uchida et al. [6] (UC16). The UM03, UM10, and UC16 models had one expression that described both the activation $(\dot{H}_A)$ and maintenance $(\dot{H}_M)$ heat rates.

Model	Work rate $(\dot{W}_{CE})$	Activation heat rate $(\dot{H}_A)$	Maintenance heat rate $(\dot{H}_M)$	Shortening heat rate $(\dot{H}_S)$	Lengthening heat rate $(\dot{H}_L)$	Scaling parameters
UM03	$F_{CE}v_M$	$f_{UM03}(P_{S,F}, a, A_{AM}), \quad \tilde{l}_M \le 1$<br>$f_{UM03}(f_l(\tilde{l}_M), P_{S,F}, a, A_{AM}), \quad \tilde{l}_M > 1$		$f_{UM03}\left(P_{S,F}, a, \frac{v_M}{l_M^0}, v_M^{MAX}, A_S\right), \quad \tilde{l}_M \le 1$<br>$f_{UM03}\left(f_l(\tilde{l}_M), P_{S,F}, a, \frac{v_M}{l_M^0}, v_M^{MAX}, A_S\right), \quad \tilde{l}_M > 1$	$f_{UM03}\left(P_{S,F}, a, \frac{v_M}{l_M^0}, v_M^{MAX}, A\right), \quad \tilde{l}_M \le 1$<br>$f_{UM03}\left(f_l(\tilde{l}_M), P_{S,F}, a, \frac{v_M}{l_M^0}, v_M^{MAX}, A\right), \quad \tilde{l}_M > 1$	m_M, s_E
BH04	$F_{CE}v_M$	$f_{BH04}(\emptyset, P_{S,F}, a_{S,F}^B)$	$f_{BH04}(\tilde{l}_M, P_{S,F}, a_{S,F}^B)$	$f_{BH04}(v_M, F_{CE}^{ISO}, F_M)$	$f_{BH04}(v_M, F_M)$	m_M
HO06	$F_{CE}v_M$	$f_{HO06}(P_{S,F}, a)$	$f_{HO06}(f_l(\tilde{l}_M), P_{S,F}, a)$	$f_{HO06}(f_l(\tilde{l}_M), P_{S,F}, a, v_M)$	*Not described*	m_M
LW07	$F_{CE}v_M$	$f_{LW07}\left(f_l(\tilde{l}_M), a, v_M^{MAX}, l_M^0, F_M^0, f_V(\tilde{v}_{CE})\right)$		$f_{LW07}(f_l(\tilde{l}_M), a, l_M^0, F_M^0, v_M)$	$0.5\ F_{CE}v_M$	
UM10	$F_{CE}v_M^{CON}$	$f_{UM03}(P_{S,F}, a, A_{AM}), \quad \tilde{l}_M \le 1$<br>$f_{UM03}(f_l(\tilde{l}_M), P_{S,F}, a, A_{AM}), \quad \tilde{l}_M > 1$		$f_{UM03}\left(P_{S,F}, a, \frac{v_M}{l_M^0}, v_M^{MAX}, A_S\right), \quad \tilde{l}_M \le 1$<br>$f_{UM03}\left(f_l(\tilde{l}_M), P_{S,F}, a, \frac{v_M}{l_M^0}, v_M^{MAX}, A_S\right), \quad \tilde{l}_M > 1$	$f_{UM03}\left(P_{S,F}, a, \frac{v_M}{l_M^0}, v_M^{MAX}, A\right), \quad \tilde{l}_M \le 1$<br>$f_{UM10}\left(f_l(\tilde{l}_M), P_{S,F}, a, \frac{v_M}{l_M^0}, v_M^{MAX}, A\right), \quad \tilde{l}_M > 1$	m_M, s_E
UC16	$F_{CE}v_M$	$f_{UM03}(P_{S,F}, a_{S,F}^U, A_{AM}), \quad \tilde{l}_M \le 1$<br>$f_{UM03}(f_l(\tilde{l}_M), P_{S,F}, a_{S,F}^U, A_{AM}), \quad \tilde{l}_M > 1$		$f_{UM03}\left(P_{S,F}, a_{S,F}^U, \frac{v_M}{l_M^0}, v_M^{MAX}, A_S\right), \quad \tilde{l}_M \le 1$<br>$f_{UM03}\left(f_l(\tilde{l}_M), P_{S,F}, a_{S,F}^U, \frac{v_M}{l_M^0}, v_M^{MAX}, A_S\right), \quad \tilde{l}_M > 1$	$f_{UM03}\left(P_{S,F}, a_{S,F}^U, \frac{v_M}{l_M^0}, v_M^{MAX}, A\right), \quad \tilde{l}_M \le 1$<br>$f_{UM03}\left(f_l(\tilde{l}_M), P_{S,F}, a_{S,F}^U, \frac{v_M}{l_M^0}, v_M^{MAX}, A\right), \quad \tilde{l}_M > 1$	m_M, s_E

Suffix S, F	Slow twitch (S) and fast twitch (F) muscle fiber, respectively
m_M	Muscle mass $[kg]$
$P_{S,F}$	Percentage of slow twitch (P_S) and fast twitch (P_F) fibers in the muscle [ ], respectively
$\tilde{l}_M, l_M^0$	Normalized fiber length [ ] and optimal fiber length $[m]$, respectively
$\tilde{v}_M, v_M, v_M^{CON}, v_M^{MAX}$	Normalized fiber velocity [ ], fiber velocity $\left[\frac{m}{s}\right]$, fiber velocity considering only concentric contraction $\left[\frac{m}{s}\right]$, and maximum contraction velocity $\left[\frac{l_M^0}{s}\right]$, respectively
$f_l(\tilde{l}_M), f_V(\tilde{v}_M)$	Force-length and force-velocity relationships [ ], respectively.
$F_M, F_{CE}, F_{CE}^{ISO}, F_M^0$	Muscle force, contractile element force, contractile element force considering only the force-length relationship and activation, and maximum force $[N]$, respectively
$\emptyset$	Decay function as described by Bhargava et al. 2004 [ ]
A_{AM}, A_S, A	Scaling factor for heat production in activation and maintenance (A_{AM}), shortening (A_S), and lengthening (A) as described by Umberger et al. 2003 [ ]
s_E	Scaling factor for aerobic conditions as described by Umberger et al. 2003 [ ]
a	Muscle activation [ ]
$a_{S,F}^B$	Muscle activation of slow twitch (a_S^B) and fast twitch (a_F^B) fibers based on orderly recruitment as described by Bhargava et al. 2004 [ ]
$a_{S,F}^U$	Muscle activation of slow twitch (a_S^U) and fast twitch (a_F^U) fibers based on active orderly recruitment as described by Uchida et al. 2016 [ ]

REFERENCES

1. Umberger BR, Gerritsen KGM, Martin PE. A model of human muscle energy expenditure. Comput Methods Biomech Biomed Engin. 2003;6: 99–111. doi:10.1080/1025584031000091678

2. Bhargava LJ, Pandy MG, Anderson FC. A phenomenological model for estimating metabolic energy consumption in muscle contraction. J Biomech. 2004;37: 81–88. doi:10.1016/S0021-9290(03)00239-2

3. Houdijk H, Bobbert MF, De Haan A. Evaluation of a Hill based muscle model for the energy cost and efficiency of muscular contraction. J Biomech. 2006;39: 536–543. doi:10.1016/j.jbiomech.2004.11.033

4. Lichtwark GA, Wilson AM. Is Achilles tendon compliance optimised for maximum muscle efficiency during locomotion? J Biomech. 2007;40: 1768–1775. doi:10.1016/j.jbiomech.2006.07.025

5. Umberger BR. Stance and swing phase costs in human walking. J R Soc Interface. 2010;7: 1329–1340. doi:10.1098/rsif.2010.0084

6. Uchida TK, Hicks JL, Dembia CL, Delp SL. Stretching your energetic budget: How tendon compliance affects the metabolic cost of running. PLoS One. 2016;11. doi:10.1371/journal.pone.0150378
