# Supplementary material for: Insights into muscle metabolic energetics: Modelling muscle-tendon mechanics and metabolic rates during walking across speeds
Source: PLoS Comput Biol. 2024 Sep 13;20(9):e1012411. doi: 10.1371/journal.pcbi.1012411 (PMC11424009; doi:10.1371/journal.pcbi.1012411)
Supplement: S2 Table — Percentage of the metabolic cost of the ankle dorsiflexors (AD), ankle plantarflexors (AP), knee flexors (KF), knee extensors (KE), hip flexors (HF), hip extensors (HE), hip adductors (HD), hip abductors (HB), hip internal rotators (HI), and hip external rotators (HO) relative to the total energy cost in a gait cycle [%] at preferred walking speed with four simulation workflows: Minimal muscle effort with generic passive force (GEN), with calibrated passive force (PAS), with calibrated passive force and personalized tendon stiffness (TEN), and EMG-informed with calibrated passive force and personalized tendon stiffness (EMG), using six metabolic energy models: Umberger et al. [1] (UM03), Bhargava et al. [2] (BH04), Houdijk et al. [3] (HO04), Lichtwark and Wilson [4] (LW07), Umberger [5] (UM10), and Uchida et al. [6] (UC16). (PDF) [file pcbi.1012411.s007.pdf]

S2 Table: Relative metabolic cost of muscle function groups at preferred walking speed. Percentage of the metabolic cost of the ankle dorsiflexors (AD), ankle plantarflexors (AP), knee flexors (KF), knee extensors (KE), hip flexors (HF), hip extensors (HE), hip adductors (HD), hip abductors (HB), hip internal rotators (HI), and hip external rotators (HO) relative to the total energy cost in a gait cycle [%] at preferred walking speed with four simulation workflows: Minimal muscle effort with generic passive force (GEN), with calibrated passive force (PAS), with calibrated passive force and personalized tendon stiffness (TEN), and EMG-informed with calibrated passive force and personalized tendon stiffness (EMG), using six metabolic energy models: Umberger et al. [1] (UM03), Bhargava et al. [2] (BH04), Houdijk et al. [3] (HO06), Lichtwark and Wilson [4] (LW07), Umberger [5] (UM10), and Uchida et al. [6] (UC16).

Simulations	Models	AD	AP	KF	KE	HF	HE	HD	HB	HI	HO
**GEN**	UM03	5.7	15.6	9.6	14.6	20.0	7.8	8.5	11.2	3.4	3.4
	BH04	7.0	20.8	10.9	12.0	17.8	7.2	7.1	10.8	3.0	3.2
	HO06	6.6	21.0	10.9	11.8	18.6	6.3	7.3	11.0	3.1	3.3
	LW07	7.8	18.6	9.3	10.7	21.3	5.8	7.4	12.1	3.7	3.3
	UM10	5.8	13.9	9.6	15.7	21.0	7.5	8.2	11.4	3.7	3.2
	UC16	6.5	18.1	10.2	12.3	20.0	7.0	7.9	11.2	3.3	3.3
**PAS**	UM03	3.4	18.3	7.9	17.2	13.3	12.6	12.5	9.1	2.0	3.6
	BH04	3.6	26.0	8.7	13.5	11.5	11.6	10.4	9.5	1.7	3.4
	HO06	3.4	26.3	8.9	15.1	11.3	11.1	10.1	9.0	1.6	3.2
	LW07	4.1	23.7	8.1	14.0	12.1	11.5	11.9	9.4	1.8	3.5
	UM10	3.6	16.9	8.0	17.8	14.1	12.7	12.0	9.2	2.2	3.7
	UC16	3.6	22.6	8.2	15.6	12.1	11.9	11.6	9.1	1.8	3.5
**TEN**	UM03	3.3	18.4	8.2	18.1	12.5	13.0	12.3	8.7	1.9	3.6
	BH04	3.7	24.2	8.7	14.7	10.8	12.6	10.7	9.2	1.7	3.5
	HO06	3.5	24.7	9.0	15.7	10.7	12.2	10.5	8.8	1.6	3.4
	LW07	4.2	23.0	8.4	13.7	11.4	12.6	12.1	9.2	1.8	3.6
	UM10	3.6	16.8	8.1	19.3	13.4	12.7	11.7	8.8	2.1	3.5
	UC16	3.6	22.3	8.5	16.5	11.3	12.5	11.5	8.7	1.7	3.5
**EMG**	UM03	6.5	16.5	8.6	14.9	13.1	14.6	12.4	7.9	2.0	3.4
	BH04	8.8	20.1	9.3	10.4	12.2	15.0	10.9	8.0	1.8	3.3
	HO06	8.4	19.8	9.4	11.6	12.6	14.6	10.8	7.8	1.8	3.3
	LW07	9.3	19.3	8.7	9.2	13.4	14.4	11.9	8.4	2.0	3.4
	UM10	6.3	16.0	8.5	16.5	14.0	13.8	11.7	7.9	2.2	3.3
	UC16	8.0	19.2	9.0	11.9	12.9	14.6	11.4	7.9	1.8	3.3

REFERENCES

1. Umberger BR, Gerritsen KGM, Martin PE. A model of human muscle energy expenditure. Comput Methods Biomech Biomed Engin. 2003;6: 99–111. doi:10.1080/1025584031000091678
2. Bhargava LJ, Pandy MG, Anderson FC. A phenomenological model for estimating metabolic energy consumption in muscle contraction. J Biomech. 2004;37: 81–88. doi:10.1016/S0021-9290(03)00239-2
3. Houdijk H, Bobbert MF, De Haan A. Evaluation of a Hill based muscle model for the energy cost and efficiency of muscular contraction. J Biomech. 2006;39: 536–543. doi:10.1016/j.jbiomech.2004.11.033
4. Lichtwark GA, Wilson AM. Is Achilles tendon compliance optimised for maximum muscle efficiency during locomotion? J Biomech. 2007;40: 1768–1775. doi:10.1016/j.jbiomech.2006.07.025
5. Umberger BR. Stance and swing phase costs in human walking. J R Soc Interface. 2010;7: 1329–1340. doi:10.1098/rsif.2010.0084
6. Uchida TK, Hicks JL, Dembia CL, Delp SL. Stretching your energetic budget: How tendon compliance affects the metabolic cost of running. PLoS One. 2016;11. doi:10.1371/journal.pone.0150378
